# Antioxidative Polyketones from the Mangrove-Derived Fungus *Ascomycota* sp. SK2YWS-L

**DOI:** 10.1038/srep36609

**Published:** 2016-11-04

**Authors:** Chunbin Tan, Zhaoming Liu, Senhua Chen, Xishan Huang, Hui Cui, Yuhua Long, Yongjun Lu, Zhigang She

**Affiliations:** 1 School of Chemistry and Chemical Engineering, Sun Yat-Sen University, Guangzhou 510275, P. R. China; 2 School of Chemistry and Environment, South China Normal University, 348 West Outer Ring Road, Guangzhou 510006, China; 3 School of Life Sciences and Biomedical Center, Sun Yat-Sen University, Guangzhou 510275, China; 4 South China Sea Bio-Resource Exploitation and Utilization Collaborative Innovation Center, Guangzhou 510006, China

## Abstract

Three novel 2,3-diaryl indone derivatives, ascomindones A−C (**1**−**3**), and two new isobenzofuran derivatives, ascomfurans A (**4**) and B (**5**), together with four know compounds (**6**−**9**) were isolated from the culture of a mangrove-derived fungus *Ascomycota* sp. SK2YWS-L. Their structures were elucidated on the interpretation of spectroscopic data. **1** and **4** were further constructed by analysis of X-ray diffraction. Antioxidant properties based on 2,2-diphenyl-1-picrylhydrazyl (DPPH), hydroxyl radical scavenging activities and the ferric reducing ability power (FRAP) of the new compounds were assayed. All of them exhibited significant effects, of which **1** showed more potent activity than ascorbic acid in scavenging DPPH radical with IC_50_ value of 18.1 *μ*M.

Overproduction of reactive oxygen species (ROS), such as hydroxyl radical and superoxide radical, playing an important role in chronic metabolic and degenerative diseases, have been proven to be specific signaling molecular under both physiological and pathophysiological conditions^1^,^2^. The balance between ROS production and antioxidant defenses determines the degree of oxidative stress, which will induce the damages of proteins, lipids and DNA in human bodies^2^. Therefore, it is important and necessary to develop effective antioxidants to keep the balance of ROS.

Mangrove-derived fungi have been demonstrated to be a rich and reliable source of biologically active and chemically unique natural products^3^,^4^,^5^,^6^. *Kandelia candel*, used as a folk medicine to cure inflammation^7^, is a typical mangrove plant with abundant fungus resources^8^ and widely distribute on the coast of South China Sea. Based on our previously researches, kinds of new compounds were obtained from the endophytic fungi of *Kanelia candel* such as the sesterterpenoids aspterpenacids A and B^9^, cyclic peptides sporothrins A, B and C^10^, polyketides 1962A and B^11^. As a part of our ongoing search for novel and bioactive metabolites from the mangrove resource, the chemical investigation of a *Kanelia candel* endophytic fungus SK2YWS−L was carried out and five new polyketones, ascomindone A−C (**1**−**3**), ascomfuran A (**4**) and B (**5**), together with four known compounds (**6**−**9**) were obtained (Fig. 1). Compounds **1**−**3** represent a novel type of 2,3-diarylindone derivatives constructing diphenyl ether or depside moiety. The details of the chemical and biological of the isolated compounds were reported herein.

## Result and Discussion

**Figure 1:**
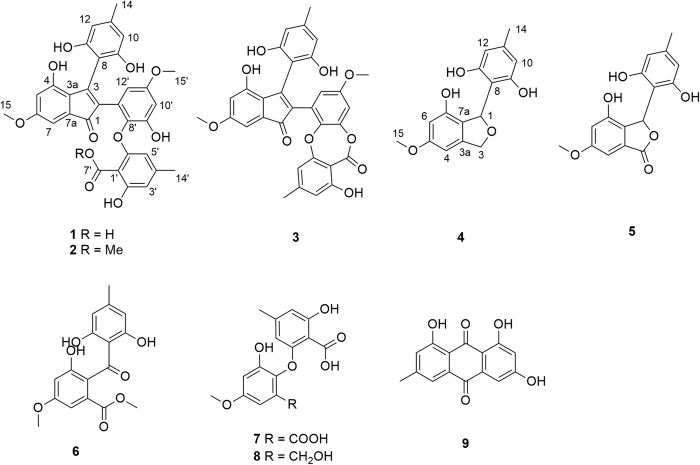
Chemical structures of compounds 1−9.

The EtOAc extract of the fermentation broth was fractionated by repeated silica gel chromatography, Sephadex LH-20 column chromatography and reversed-phase C_18_ semipreparative High Performance Liquid Chromatography (HPLC) to afford the five new compounds (**1**−**5**) together with the three known compounds methyl 2-(2,6-dihydroxy-4-methylbenzoyl)-3-hydroxy-5-methoxybenzoate (**6**), 2-(2-carboxy-3-hydroxy-5-methylphenoxy)-3-hydroxy-5-methoxybenzoic acid (**7**)^12^, 2-hydroxy-6-(2-hydroxy-6-(hydroxymethyl)-4-methoxyphenoxy)-4-methylbenzoic acid (**8**) and emodin (**9**)^13^. The structures of the new compounds were deduced by spectroscopic data as well as X-ray crystallographic analysis and the identifications of the known compounds were based on the comparison of their spectroscopic data with those previously reported (Fig. 1).

**Figure 2:**
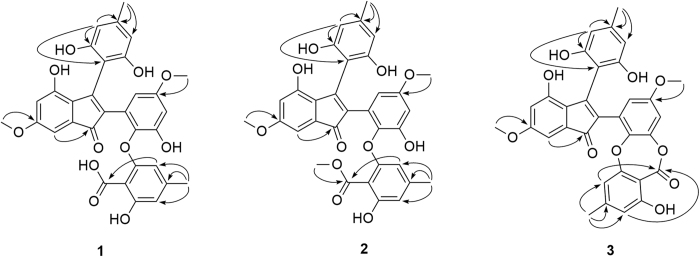
Key HMBC correlations (arrows) of compounds 1−3.

Ascomindone A (**1**) was obtained as brown crystals. The molecular formula was determined as C_32_H_26_O_11_ based on the quasi-molecular ion peak at *m/z* 585.1401 ([M − H]^−^, calcd for 586.1475) from HRESIMS, indicating 20 degrees of unsaturation. The IR spectrum exhibited the absorption bands of hydroxyl groups (3425 and 3219 cm^−1^) and carbonyl groups (1636 and 1670 cm^−1^). The ^1^H NMR (Nuclear Magnetic Resonance) spectrum (Table 1) revealed the presence of a chelating hydroxyl group at *δ*_H_ 9.31 (1H, s), eight aromatic protons at *δ*_H_ 5.92 (H-3′, brs), *δ*_H_ 6.09 (H-10 and H-12, brs), *δ*_H_ 6.24 (H-5, d, *J* = 2.2 Hz), *δ*_H_ 6.34 (H-10′, d, *J* = 3.1 Hz), *δ*_H_ 6.34 (H-5′, brs), *δ*_H_ 6.38 (H-12′, brs) and *δ*_H_ 6.51 (H-7, d, *J* = 2.2 Hz), two methoxyl groups at *δ*_H_ 3.54 (H_3_-15′, s) and 3.71 (H_3_-15, s), two methyls at *δ*_H_ 2.09 (H_3_-14′, s) and 2.12 (H_3_-14, s). ^13^C NMR and DEPT spectra (Table 1) resolved 32 carbon resonances composed of four sp^3^ hybrid methyl carbons, two carbonyl carbons and 26 aromatic carbons including eight methines and 18 quaternary carbons. A comprehensive analysis of 1D NMR data demonstrated the presences of four *tetra*-substituted benzene rings and a conjugated double bond in **1**, of which a symmetric benzene ring was deduced by the HMBC correlations (Fig. 2) from the pair of chemically equivalent protons (H-10 and H-12) to C-8, C-9 (C-13) and C-11. The chemical shift of the ketone carbon (*δ*_C_ 194.1) and the degrees of unsaturation suggested there should be an 2,3-disubstituded indone moiety in compound **1**, which was further evidenced by the correlation from H-7 to C-1 in HMBC spectrum. The remaining two *tetra*-substituted benzene rings should be incorporated into a diphenyl ether moiety based on their corresponding ^13^C chemical shifts. Additionally, the placements of the hydroxyl, methyl and methoxyl groups were unambiguously elucidated by the chemical shifts and the HMBC correlations (Fig. 2) from H_3_-14 to C-10/C-11/C-12, H_3_-14′ to C-3′/C-4′/C-5′, H_3_-15 to C-6, and H_3_-15′ to C-11′, while the carboxyl group was located at C-1′ based on a weak four-bond correlation from H-5′ to C-7′.

**Figure 3:**
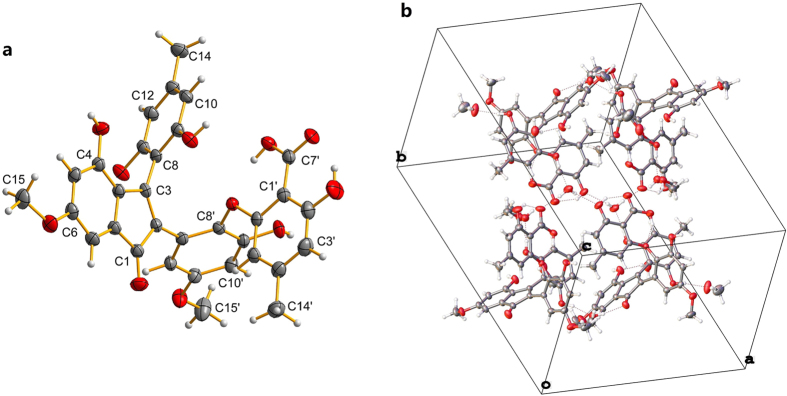
Single-crystal X-ray structure (**a**) and molecular packing properties (**b**) of compound 1.

However, the connectivity of the above fragments was still difficult to deduce since the lack of HMBC correlations in such a complex structure^14^. Fortunately, the complete structure was finally confirmed by the X-ray crystallographic analysis as shown in Fig. 3. Ascomindone A (**1**) was an axially chiral biaryl natural product, which should have had the specific optical rotation and Cotton effects (CEs) in Circular Dichroism (CD) spectrum. However, the lack of any specific optical rotation or significant CEs indicated that **1** was a racemic mixture of *M*- and *P*-helicity enantiomers with the ratio of 1:1. Further analysis of the molecular packing in crystals presented the mixture of (±)**-1** (Fig. 3).

Ascomindone B (**2**), isolated as brown powder, was deduced to possess the molecular formula of C_33_H_28_O_11_ based on the HRESIMS at *m/z* 599.1558 ([M − H]^−^, calcd for 600.1631). Analysis of the 1D NMR spectra suggested that compound **2** was structurally similar to **1** except for the presence of an additional methoxyl group. The HMBC correlation from OMe-7′ (*δ*_H_ 3.64) to the carbonyl carbon (C-7′) indicated that the methoxyl was linked to C-7′. The gross structure was elucidated based on 2D NMR data (Fig. 2).

Ascomindone C (**3**) was obtained as brown powder and showed the molecular formula of C_32_H_26_O_11_ based on the HRESIMS at *m/z* 567.1298 ([M − H]^−^, calcd for 568.1369), suggesting that **3** was the dehydration product of **1**. Comparison of 1D and 2D NMR with those of **1** indicated that **3** constructed the same 2,3-disubstituded indanone and the symmetric *tetra*-substituted benzene ring moieties. The major difference between them was that the diphenyl ether moiety in **1** was transformed to a depside in **3** through intramolecular esterification, which was confirmed by the upfield shift (∆*δ*_C_ = 7.8 ppm) of the carbonyl carbon (C-7′) in **3**. The complete structure of **3** was deduced by the HMBC spectra (Fig. 2) as shown in Fig. 1.

Ascomindones B and C (**2** and **3**) were both inferred to be racemates since the specific optical rotation were zero and there were no distinct CEs in CD data.

Ascomfuran A (**4**) was isolated as yellow crystals. HRESIMS analysis afforded an [M − H]^−^ ion peak at *m/z* 287.0927, indicating the molecular formula as C_16_H_16_O_5_. The IR data exhibited absorption of hydroxyl (3395 cm^−1^) functionality. The ^1^H NMR spectrum (Table 2) revealed presences of a set of *meta*-coupled aromatic protons at *δ*_H_ 6.34 (H-4, d, *J* = 1.9 Hz) and *δ*_H_ 6.20 (H-6, d, *J* = 1.9 Hz), a pair of chemically equivalent aromatic protons at *δ*_H_ 6.16 (H-10 and H-12, s), a couple of oxygenated methylene in AB spin system protons at *δ*_H_ 5.33 (H-3*α*, dd, *J* = 2.7, 11.6 Hz) and *δ*_H_ 5.00 (H-3*β*, d, *J* = 11.6 Hz), a methoxyl signal at *δ*_H_ 3.73 (H_3_-15, s) and a methyl signal at *δ*_H_ 2.16 (H_3_-14, s). ^13^C NMR and DEPT spectra exhibited 16 carbon resonances, containing eight quaternary carbons, five methines, one methylene and two methyls. A comprehensive analysis of the 1D NMR data suggested that there should be two *tetra*-substituted benzene rings including a symmetric one.

**Figure 4:**
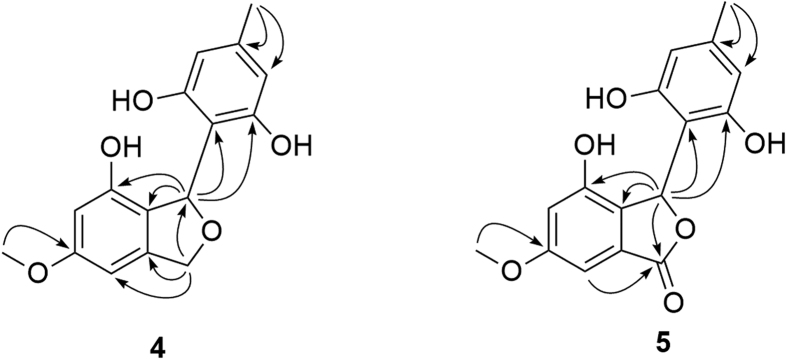
Key HMBC correlations (arrows) of compounds 4 and 5.

The HMBC correlations (Fig. 4) from H_2_-3 to C-3a/C-4/C-7a/C-1 and from H-1 to C-3/C-7/C-7a, combined with the degrees of unsaturation indicated an isobenzofurane moiety in **4**. The symmetric benzene moiety was connected to C-1 based on the HMBC correlations from H-1 to C-8/C-9(C-13). In addition, the cross-peaks of H_3_-15 to C-5 and H_3_-14 to C-10(C-12)/C-11 were detected, which revealed the attachments of the methoxyl and the methyl group at C-5 and C-11, respectively. Thus, the planar structure of **4** was constructed as shown in Fig. 1.

**Figure 5:**
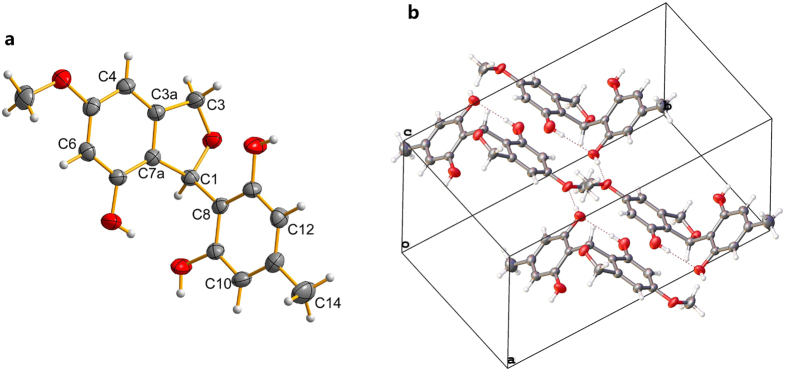
Single-crystal X-ray structure (**a**) and molecular packing properties (**b**) of compound 4.

Given the lack of any specific optical rotation or significant Cotton effects in CD spectrum, **4** was deduced to be racemic mixtures at C-8. A further X-ray diffraction experiment was carried out and the enantiomers were performed in molecular packing of crystal (Fig. 5).

Ascomfuran B (**5**), isolated as yellowish amorphous power, was assigned a molecular formula of C_16_H_14_O_6_ based on the HREI-MS *m/z* 302.0790 ([M − e]^+^, calcd for C_16_H_14_O_6_ 302.0785). The IR spectrum exhibited hydroxyl (3212 cm^−1^) and additional carbonyl (1702 cm^−1^) functional groups. ^1^H NMR data were similar to those of ascomfuran A (**4**), except for the absence of the methylene signals. In the ^13^C NMR spectrum, the disappearance of the corresponding methylene signal and the presence of an additional carbonyl carbon signal at *δ*_C_ 173.5 suggested that the methylene in **4** was oxidized to a carbonyl group in **5**, which was confirmed by the HMBC correlations (Fig. 4) from H-1 and H-4 to C-3. Hence, the planar structure was deduced as shown in Fig. 1. Compound **5** was also obtained as a racemate at C-1 based on the weak specific optical rotation or Cotton effects in CD spectrum.

**Figure 6:**
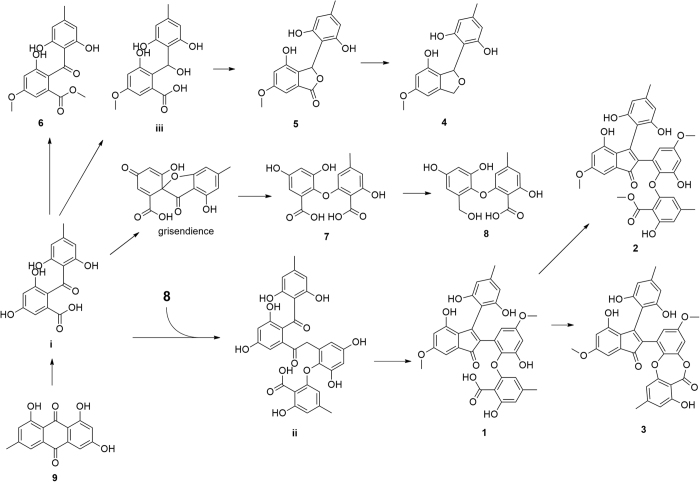
Plausible biogenetic pathway of compounds 1−5.

The hypothetical biosynthesis pathways of **1**−**5** were proposed in Fig. 6. The oxidation of emodin (**9**) gave the benzophenone intermediate (structure **i**), which could further transform into diphenyl ether derivatives (**7** and **8**) via grisendience^15^,^16^. Generated by **i** and **8**^17^, intermediate **ii** could afford compound **1**−**3** through an aldol condensation as well as the additional esterification. In addition, **4** and **5** were transformed from intermediate **iii** by esterification and reduction, which was a reductive product of intermediate **i**.

To the best of our knowledge, 2,3-diarylindone derivatives are quite rare in natural products^14^ Ascomindones A−C (**1**−**3**), represent the first examples of 2,3-diarylindone derivatives constructing diphenyl ether or depside moiety. It is challenging to elucidate such a class of structures with low H/C ratio using NMR spectroscopic methods based on the Crews’s rule^18^. However, through the extensive NMR experiments and X-ray crystallographic analysis, the structures of **1**−**3** were deduced unambiguously. In addition, ascomfurans A (**4**) and B (**5**), belong to the derivatives of 1-aryl isobenzofuran, were naturally obtained racemic mixture like isopestacin and pestacin since the enantiomers of 1*S* and 1*R* could transform to each other through a stable cationic intermediate^19^,^20^.

**Figure 7:**
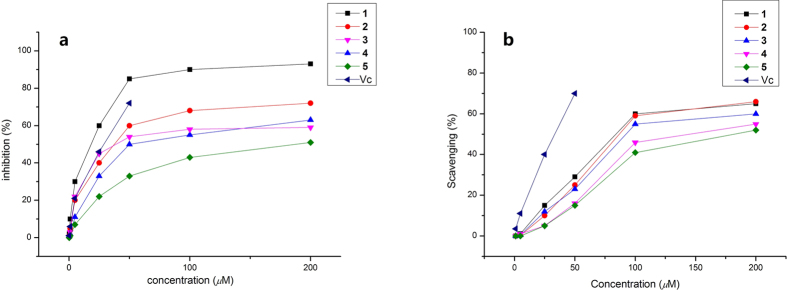
DPPH radical (**a**) and hydroxyl radical (**b**) scavenging capacity of compounds **1−5**.

**Figure 8:**
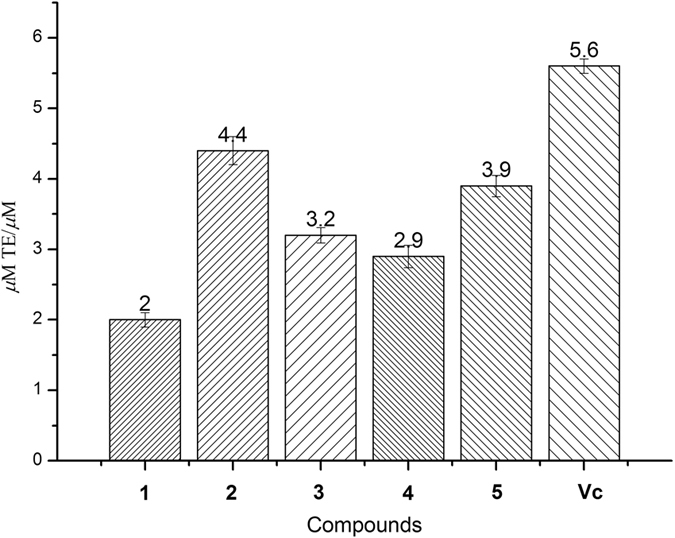
Antioxidant capacity of compounds 1−5 as determined by FRAP.

Naturally, phenolic compounds are proven to be the effective antioxidants^21^,^22^. Hence, all the isolated compounds were evaluated for their *in vitro* antioxidative activities based on 2,2-diphenyl-1-picrylhydrazyl (DPPH), hydroxyl radical scavenging capacities and ferric reducing ability power (FRAP) assays. Compared to the positive control ascorbic acid (Vc), ascomindone A (**1**) exhibited more potent capacity in scavenging DPPH radical with a IC_50_ value of 18.1 *μ*M while compounds **2**−**4** also showed significant effect (Fig. 7). In hydroxyl radical scavenging assay, ascomindones A−C (**1**−**3**) exhibited strong activity with the IC_50_ values in the range from 80 to 100 *μ*M (Fig. 7). In addition, compound **1**−**5** also showed potent activity in FRAP assay as shown in Fig. 8.

## Methods

### General experimental procedures

Melting points were determined on a Fisher-Johns hot-stage apparatus and were uncorrected. UV data were measured on a UV-240 spectrophotometer (Shimadzu, Beijing, China). HRMS (ESI) were determined with a Q-TOF high-resolution mass spectrometer (Waters). HREIMS data were measured on a MAT95XP high-resolution mass spectrometer (Thermo). IR spectrum was recorded using Bruker Vector spectrophotometer 22. Optical rotation was recorded using a an MCP300 (Anton Paar, Shanghai, China). CD data were recorded with a J-810 spectropolarimeter (JASCO, Tokyo, Japan). The NMR data were recorded on a Bruker Avance 600 spectrometer (Bruker, Beijing, China) at 600 MHz for ^1^H and 125 MHz for ^13^C, respectively. All chemical shifts (*δ*) are given in ppm with reference to TMS, and coupling constants (J) are given in Hz. Column chromatography (CC) was carried out on silica gel (200–300 mesh, Marine Chemical Factory, Qingdao, China) and sephadex LH-20 (Amersham Pharmacia, Piscataway, NJ, USA). Solvents were distilled prior to use. Semipreparative HPLC was performed on a Waters Breeze HPLC system using a Phenomenex Luna (Phenomenex, Torrance, CA, USA) C18 column (250 × 10 mm, 5 *μ*m), flow rate, 2.0 mL/min.

### Fungal material

The fungus used in this study was isolated from the healthy leaf of the marine mangrove *Kandelia candel*, which were collected in April 2012 from Shankou Mangrove Nature Reserve in Guangxi Province, China. It was obtained using the standard protocol for the isolation. Fungal identification was carried out using a molecular biological protocol by DNA amplification and sequencing of the ITS region. The sequence data obtained from the fungal strain have been deposited at GenBank with accession no. KX389270. A BLAST search result showed that the sequence was the most similar (100%) to the sequence of *Ascomycota* sp. (compared to KC857277.1 HQ647349.1). A voucher strain was deposited in School of Chemistry and Chemical Engineering, Sun Yat-Sen University, Guangzhou, China.

### Fermentation, extraction and isolation

The fungus was grown on liquid cultured medium (composed of maltose (20.0 g/L), mannitol (20.0 g/L), glucose (10.0 g/L), monosodium glutamate (10.0 g/L), MgSO_4_•7H_2_O (0.3 g/L), KH_2_PO4 (0.5 g/L), yeast extract (3.0 g/L), corn steep liquor (1.0 g/L)) in 80 Erlenmeyer flasks for 45 days at room temperature under static condition. After fermentation, the former was extracted with EtOAc and concentrated under reduced pressure to yield residual gum in 5.4 g. The residue was subjected to silica gel CC using gradient elution with petroleum ether-EtOAc from 90:10 to 0:100 (v/v) to give twelve fractions (Frs.1–10). Fr. 5 (207 mg) was further purified by silica gel CC using CHCl_3_/MeOH (99:1) to obtain **4** (3.5 mg), **5** (3.3 mg), **6** (9.2 mg), **7** (9.8 mg) and **8** (11.9 mg) Fr. 8 (117 mg) was further purified by silica gel CC using CHCl_3_/MeOH (97:3) to afford six subfractions (Frs.8.1-8.6). Fr. 8.5 (20.1 mg) was applied to Sephedx LH-20 CC, eluted with MeOH to obtain **1** (10.8 mg) and **9** (8.3 mg). After purification by RP-HPLC (80% MeOH in H_2_O for 5 min, followed by 80–100% over 30 min; 1.5 mL/min), Fr. 8.2 (30.2 mg) afforded **2** (5.4 mg, *t*_R_ = 9.3 min) and **3** (7.2 mg, *t*_R_ = 11.9 min).

#### Ascomindone A (**1**)

brown crystal; *m.p.* 301 ~ 302 °C; [*α*]_D_ = 0 (0.1 M in methanol); UV (MeOH): *λ*_max_: 275, 382 nm. IR (KBr): 3425, 3219, 1670, 1636, 1430, 1278, 1199, 1055 cm^−1^; HRESIMS *m/z* 585.1401 [M − H]^−^ (calcd for C_32_H_26_O_11_ 586.1475); ^1^H and ^13^C NMR data: see Table 1 and supplementary information.

#### Ascomindone B (**2**)

yellowish powder; *m.p.* 277 ~ 279 °C; [*α*]_D_ = 0 (0.1 M in methanol); UV (MeOH) *λ*_max_: 275, 358 nm. IR (KBr): 3391, 1695, 1619, 1430, 1303, 1148, 1063 cm^−1^; HRESIMS *m/z* 599.1558 [M − H]^−^ (calcd for C_33_H_28_O_11_ 600.1631); ^1^H and ^13^C NMR data: see Table 1 and supplementary information.

#### Ascomindone C (**3**)

yellowish powder; *m.p.* 279 ~ 281 °C; [*α*]_D_ = 0 (0.1 M in methanol); UV (MeOH) *λ*_max_: 275, 360 nm. IR (KBr): 3408,1687, 1610, 1455, 1190, 1131, 1055 cm^−1^; HRESIMS m/z 567.1298 [M − H]^−^ (calcd for C_32_H_26_O_11_ 568.1369); ^1^H and ^13^C NMR data: see Table 1 and supplementary information.

#### Ascomfuran A (**4**)

yellowish crystal; *m.p.* 218 ~ 220 °C; [*α*]_D_ = 0 (0.1 M in methanol); UV (MeOH) *λ*_max_: 245 nm. IR (KBr): 3395, 2922, 2850, 1459, 1346, 1202, 1149 cm^−1^; HRESIMS *m/z* 287.0927 [M − H]^−^ (calcd for C_16_H_16_O_5_ 288.0998); ^1^H and ^13^C NMR data: see Table 2 and supplementary information.

#### Ascomfuran B (**5**)

yellowish powder; *m.p.* 232 ~ 233 °C. [*α*]_D_ = 0 (0.1 M in methanol). UV (MeOH) *λ*_max_: 252, 284 nm. IR (KBr): 3411, 3212, 2968, 1702, 1416, 1303, 1209, 1154, 1055 cm^−1^. HREIMS *m/z* 302.0790 [M]^+^ (calcd for C_16_H_14_O_6_ 302.0785); ^1^H and ^13^C NMR data: see Table 2 and supplementary information.

### Crystallographic Data and X-ray Analysis

Brown crystals of ascomindone A (**1**) were obtained from MeOH containing a small amount of H_2_O at room temperature. Data were collected on Agilent Xcalibur Nova single-crystal diffractometer using Cu K*α* radiation. The crystal structure was refined by full-matrix least-squares calculation with the SHELXL-97. Crystallographic data for the structure of **1** have been deposited in the Cambridge Crystallographic Data Centre (deposition number: CCDC 1483330). Crystal data of **1**: C_32_H_26_O_11_∙2CH_3_OH∙H_2_O (M = 668.63); block crystal (0.4 × 0.4 × 0.38); space group *P*21/c; unit cell dimensions *a* = 17.5917(5) Å, *b* = 25.5009(10) Å, *c* = 14.2645(5) Å, *α* = 90°, *β* = 93.021(3)°, *γ* = 90°, *V* = 6390.2(4) Å^3^, *Z* = 8; *T* = 150(2) K; *ρ*_cald_ = 1.390 mg/m^3^; absorption coefficient 0.918 mm^−1^; *F*(000) = 2816, a total of 11658 reflections were collected in the range 3.05° < θ < 68.67°, independent reflections 9974 [*R*(int) = 0.0334]; the number of data/parameters/restraints were 11658/896/0; goodness-offit on *F*^2^ = 1.024; final *R* indices [*I* > 2*σ*(*I*)] *R*_1_ = 0.0438, *ωR*_2_ = 0.1156; *R* indices (all data) *R*_1_ = 0.0528, *ωR*_2_ = 0.1230.

Yellow crystals of ascomfuran A (**4**) were obtained from MeOH containing a small amount of H_2_O at room temperature. Data were collected on Agilent Xcalibur Nova single-crystal diffractometer using Mo K*α* radiation. The crystal structure was refined by full-matrix least-squares calculation with the SHELXL-97. Crystallographic data for the structure of **1** have been deposited in the Cambridge Crystallographic Data Centre (deposition number: CCDC 1483331). Crystal data of **1**: C_16_H_16_O_5_ (M = 288.29); block crystal (0.4 × 0.4 × 0.35); space group *P*21/c; unit cell dimensions *a* = 9.6005(6) Å, *b* = 18.5783(9) Å, *c* = 8.3575(5) Å, *α* = 90°, *β* = 101.567(6)°, *γ* = 90°, *V* = 1460.38(14) Å^3^, *Z* = 4; *T* = 293(2) K; *ρ*_cald_ = 1.311 mg/m^3^; absorption coefficient 0.098 mm^−1^; *F*(000) = 608, a total of 3253 reflections were collected in the range 3.67° < θ < 27.40°, independent reflections 2532 [*R*(int) = 0.0467]; the number of data/parameters/restraints were 3253/195/0; goodness-offit on *F*^2^ = 1.097; final *R* indices [*I* > 2*σ*(*I*)] *R*_1_ = 0.0607, *ωR*_2_ = 0.1296; *R* indices (all data) *R*_1_ = 0.0826, *ωR*_2_ = 0.1428.

### DPPH radical scavenging activity assay

The DPPH radical scavenging test was based on the previous reported method^23^ but with slight modification. The activity test was performed in 96-well microplates. A range of 50 *μ*L solutions of different concentrations (2, 25, 50, 100, 200 *μ*M) of the tested compounds **1**−**5** was added to 150 *μ*L (0.16 mmol/L) DPPH solution in MeOH in each well. Absorbance at 517 nm was recorded after 45 min and the percentage of inhibition was calculated. Vitamin C was used as a positive control.

### Hydroxyl radical scavenging activity assay

The hydroxyl radical-scavenging assay was determined based on the described in previous reports^24^. PMPH fraction (15 *μ*L) was mixed with 25 *μ*L of FeSO_4_ solution (3 mM) and 25 *μ*L of 1,10-phenanthroline (3 mM, dissolved in 0.1 M phosphate buffer, pH = 7.4). Furthermore, 0.01% (v/v) H_2_O_2_ peroxide (25 *μ*L) was added into the mixture. After incubated at 37 °C for 1 h, the absorbance was measured at 536 nm.

### The FRAP assay

The FRAP assay performed was slight modified according to the previous reported literature^25^,^26^,^27^. The FRAP reagent was freshly prepared by adding 3 M CH_3_COOH buffer (pH 3.6), 0.1 M 2,4,6-Tris(2-pyridyl)-s-triazine and 0.2 M FeCl3 at 10:1:1 volume ratio in 0.4 M HCl. 180 *μ*L FRAP reagent and 20 *μ*L tested compound (100 *μ*M) were added in 96-well microplates. After incubated at 37 °C for 20 min, the absorbance of the mixture was meansured at 595 nm. Vitamin C was used as a positive control. The FRAP value was expressed in Trolox (a water-soluble analog of vitamin E) equivalents using the linear slope of the compounds tested versus that of Trolox.

## Additional Information

**How to cite this article**: Tan, C. *et al*. Antioxidative Polyketones from the Mangrove-Derived Fungus *Ascomycota* sp. SK2YWS-L. *Sci. Rep.* **6**, 36609; doi: 10.1038/srep36609 (2016).

**Publisher’s note:** Springer Nature remains neutral with regard to jurisdictional claims in published maps and institutional affiliations.

## Supplementary Material

Supplementary Information

## Figures and Tables

**Table 1:** ^1^H (600 MHz) and ^13^C (150 MHz) NMR Data of 1−3.

Position	1^a^		2^a^		3^b^	
	*δ*_C_, type	*δ*_H_, m (*J* in Hz)	*δ*_C_, type	*δ*_H_, m (*J* in Hz)	*δ*_C_, type	*δ*_H_, m (*J* in Hz)
1	194.1, C		193.7, C		193.9, C	
2	129.4, C		130.4, C		134.2, C	
3	156.9, C		155.6, C		152.9, C	
3a	120.8, C		121.2, C		121.5, C	
4	152.7, C		152.7, C		153.2, C	
5	106.1, CH	6.24, d (2.1)	105.9, CH	6.24, d (2.1)	107.2, CH	6.32, d (2.1)
6	161.3, C		161.2, C		162.9, C	
7	102.2, CH	6.51, d (2.1)	102.0, CH	6.48, d (2.1)	103.9, CH	6.64, d (2.1)
7a	133.9, C		134.3, C		134.4, C	
8	107.4, C		107.6, C		112.8, C	
9	155.1, C		155.2, C		148.3, C	
10	106.7, CH	6.09, brs	106.7, CH	6.07, brs	115.4, CH	6.71, brs
11	138.2, C		138.0, C		140.4, C	
12	106.7, CH	6.09, brs	106.7, CH	6.07, brs	113.6, CH	6.64, brs
13	155.1, C		155.2, C		157.2, C	
14	21.2, CH_3_	2.12, s	21.3, CH_3_	2.10, s	21.5, CH_3_	2.18, s
15	55.5, CH_3_	3.71, s	55.6, CH_3_	3.71, s	56.0, CH_3_	3.74, s
1′	109.5, C		106.6, C		109.5, C	
2′	158.0, C		156.2, C		158.3, C	
3′	107.1, CH	5.92, brs	109.0, CH	6.22, brs	112.8, CH	6.50, brs
4′	144.5, C		141.2, C		142.5, C	
5′	110.5, CH	6.34, brs	106.5, CH	5.78, brs	110.6, CH	6.38, brs
6′	160.1, C		156.8, C		156.7, C	
7′	170.6, C		168.0, C		162.8, C	
8′	133.7, C		133.9, C		137.4, C	
9′	149.5, C		149.7, C		150.5, C	
10′	102.4, CH	6.33, d (3.1)	102.3, CH	6.32, d (3.0)	103.6, CH	6.27, d (3.0)
11′	156.1, C		155.7, C		157.5, C	
12′	105.2, CH	6.38, brs	105.3, CH	6.37, d (3.0)	106.8, CH	5.82, d (3.0)
13′	127.9, C		128.1, C		129.3, C	
14′	21.6, CH_3_	2.09, s	21.6, CH_3_	2.06, s	21.3, CH_3_	2.23, s
15′	54.8, CH_3_	3.54, s	54.8, CH_3_	3.52, s	55.3, CH_3_	3.46, s
OMe−7′	—	—	51.7	3.64, s	—	—

^a^meansured in DMSO-*d*_6_.

^b^meansured in acetone-*d*_6_.

**Table 2:** ^1^H (500 MHz) and ^13^C (125 MHz) NMR Data of 4 and 5.

Position	**4**^a^		**5**^a^	
	*δ*_C_, type	*δ*_H_, m (*J* in Hz)	*δ*_C_, type	*δ*_H_, m (*J* in Hz)
1	79.6, CH	6.72, s	77.1, CH	6.84, s
3	74.4, CH_2_	5.33, dd (2.7, 11.6)	173.5, C	
		5.00, d (11.6)		
3a	143.1, C		168.2, C	
4	98.2, CH	6.34, d (1.9)	102.0, CH	6.34, d (1.6)
5	162.8, C		168.2, C	
6	101.8, CH	6.20, d (1.9)	99.2, C	6.23, brs
7	153.5, C		157.0, C	
7a	120.3, C		107.4, C	
8	111.4, C		107.3	
9	157.1, C		158.7, C	
10	109.4, CH	6.16, s	108.6, CH	6.13, s
11	140.3, C		142.1, C	
12	109.4, CH	6.16, s	108.6, CH	6.13, s
13	157.1, C		157.0, C	
14	21.3, CH_3_	2.16, s	21.5, CH_3_	2.16, s
15	55.9, CH_3_	3.73, s	56.2, CH_3_	3.76, s

^a^meansured in methanol-*d*_4_.
