# Supplementary material for: Antioxidative Polyketones from the Mangrove-Derived Fungus Ascomycota sp. SK2YWS-L
Source: Sci Rep. 2016 Nov 4;6:36609. doi: 10.1038/srep36609 (PMC5095669; doi:10.1038/srep36609)
Supplement: Supplementary Information [file srep36609-s1.pdf]

Supporting information

Antioxidative Polyketones from the Mangrove-Derived Fungus *Ascomycota* sp. SK2YWS-L

Chunbin Tan[1†], Zhaoming Liu[1†], Senhua Chen[1], Xishan Huang, Hui Cui[1], Yuhua Long[1,2,*], Yongjun Lu[3,4] and Zhigang She[1, 4,*]

[1]*School of Chemistry and Chemical Engineering, Sun Yat-Sen University, Guangzhou 510275, P. R. China;*
[2]*School of Chemistry and Environment, South China Normal University, 348 West Outer Ring Road, Guangzhou 510006, China;*
[3]*School of Life Sciences and Biomedical Center, Sun Yat-Sen University, Guangzhou 510275, China;*
[4]*South China Sea Bio-Resource Exploitation and Utilization Collaborative Innovation Center, Guangzhou 510006, China.*

[*]Corresponding author: Y.L. (yuhualong68@hotmail.com) or Z.S. (cesshzhg@mail.sysu.edu.cn)
[†]T.C. and L.Z. contributed equally to this work.

Table of contents:

Figure S1. ^{1}H NMR spectrum of compound **1** in DMSO-d_6 (600 MHz).
Figure S2. ^{13}C NMR spectrum of compound **1** in DMSO-d_6 (150 MHz).

Figure S3. DEPT-90 °spectrum of compound **1** in DMSO-d_6 (150 MHz).

Figure S4. DEPT-135 °spectrum of compound **1** in DMSO-d_6 (150 MHz).

Figure S5. ^{1}H-^{1}H COSY spectrum of compound **1** in DMSO-d_6 (600 MHz).

Figure S6. HSQC spectrum of compound **1** in DMSO-d_6 (600 MHz).

Figure S7. HMBC spectrum of compound **1** in DMSO-d_6 (600 MHz).

Figure S8. ^{1}H NMR spectrum of compound **2** in DMSO-d_6 (600 MHz).

Figure S9. ^{13}C NMR spectrum of compound **2** in DMSO-d_6 (150 MHz).

Figure S10. DEPT-90 °spectrum of compound **2** in DMSO-d_6 (150 MHz).

Figure S11. DEPT-135 °spectrum of compound **2** in DMSO-d_6 (150 MHz).

Figure S12. ^{1}H-^{1}H COSY spectrum of compound **2** in DMSO-d_6 (600 MHz).

Figure S13. HSQC spectrum of compound **2** in DMSO-d_6 (600 MHz).

Figure S14. HMBC spectrum of compound **2** in DMSO-d_6 (600 MHz).

Figure S15. ^{1}H NMR spectrum of compound **3** in acetone-d_6 (600 MHz).

Figure S16. ^{13}C NMR spectrum of compound **3** in acetone-d_6 (150 MHz).

Figure S17. DEPT-90 °spectrum of compound **3** in acetone-d_6 (150 MHz).

Figure S18. DEPT-135 °spectrum of compound **3** in acetone-d_6 (150 MHz).

Figure S19. ^{1}H-^{1}H COSY spectrum of compound **3** in acetone-d_6 (600 MHz).

Figure S20. HSQC spectrum of compound **3** in acetone-d_6 (600 MHz).

Figure S21. HMBC spectrum of compound **3** in acetone-d_6 (600 MHz).

Figure S22. ^{1}H NMR spectrum of compound **4** in methanol-d_4 (500 MHz).

Figure S23. ^{13}C NMR spectrum of compound **4** in methanol-d_4 (125 MHz).

Figure S24. DEPT-90 °spectrum of compound **4** in methanol-d_4 (125 MHz).

Figure S25. DEPT-135 °spectrum of compound **4** in methanol-d_4 (125 MHz).

Figure S26. ^{1}H-^{1}H COSY spectrum of compound **4** in methanol-d_4 (500 MHz).

Figure S27. HSQC spectrum of compound **4** in methanol-d_4 (500 MHz).

Figure S28. HMBC spectrum of compound **4** in methanol-d_4 (500 MHz).

Figure S29. ^{1}H NMR spectrum of compound **5** in methanol-d_4 (500 MHz).
Figure S30. ^{13}C NMR spectrum of compound **5** in methanol-d_4 (125 MHz).
Figure S31. DEPT-90 °spectrum of compound **5** in methanol-d_4 (125 MHz).
Figure S32. DEPT-135 °spectrum of compound **5** in methanol-d_4 (125 MHz).
Figure S33. ^{1}H-^{1}H COSY spectrum of compound **5** in methanol-d_4 (500 MHz).
Figure S34. HSQC spectrum of compound **5** in methanol-d_4 (500 MHz).
Figure S35. HMBC spectrum of compound **5** in methanol-d_4 (500 MHz).
Figure S36. HRESIMS spectrum of compound **1**.
Figure S37. HRESIMS spectrum of compound **2**.
Figure S38. HRESIMS spectrum of compound **3**.
Figure S39. HRESIMS spectrum of compound **4**.
Figure S40. HREI-MS spectrum of compound **5**.

Figure S1. ^{1}H NMR spectrum of compound **1** in DMSO-d_6 (600 MHz).

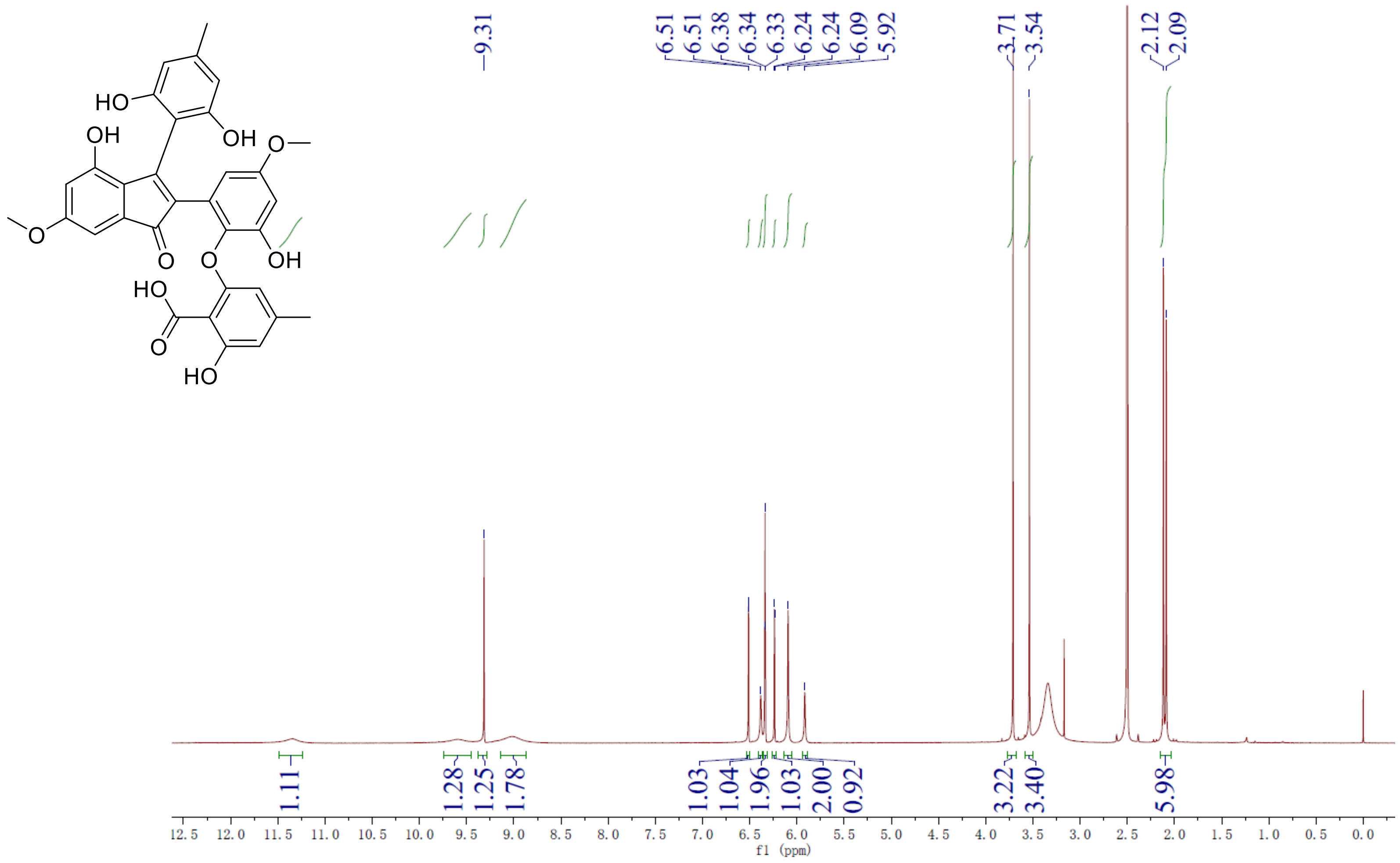

Figure S2. ^{13}C NMR spectrum of compound **1** in DMSO-d_6 (150 MHz).

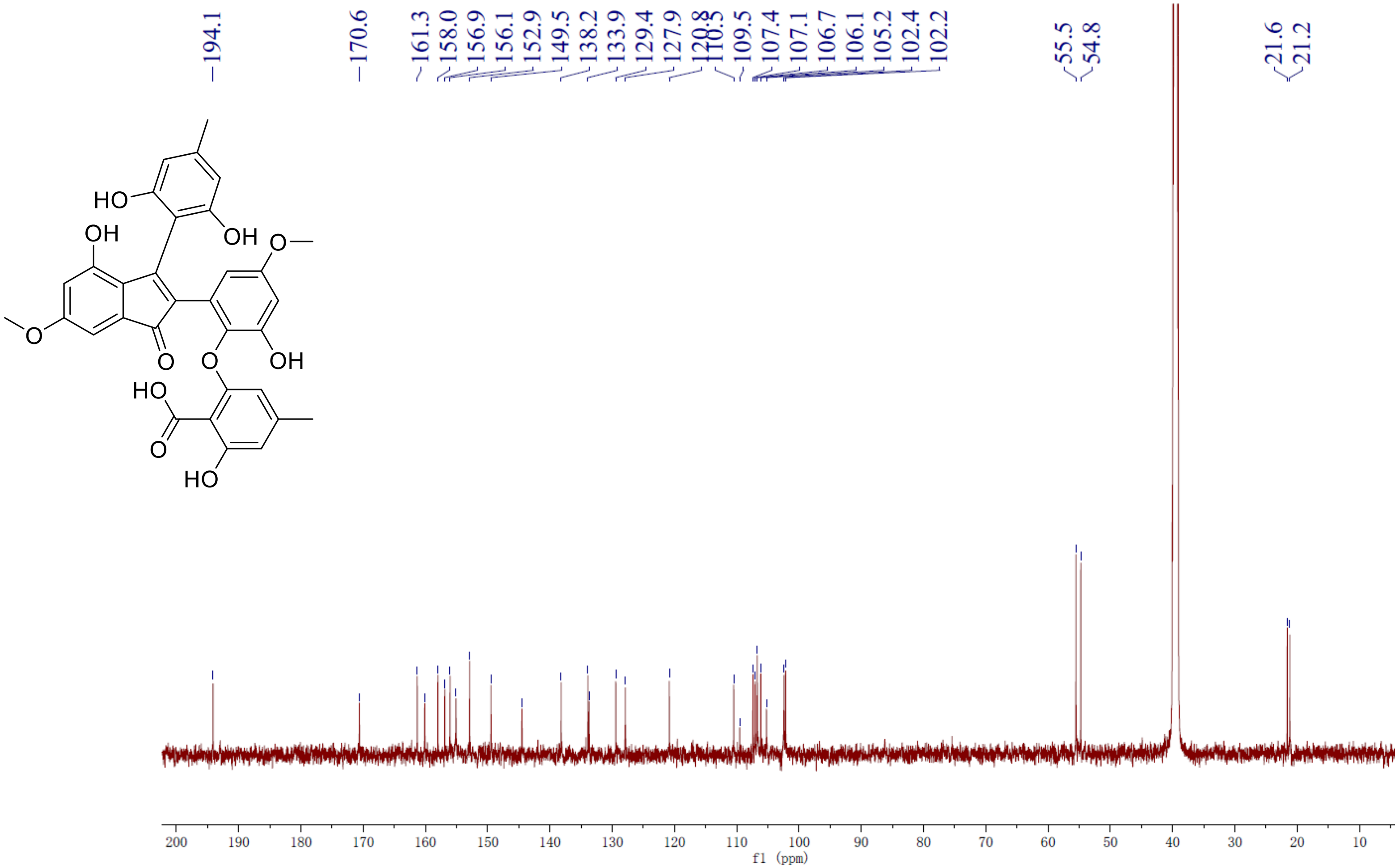

Figure S3. DEPT-90 °spectrum of compound **1** in DMSO-d_6 (150 MHz).

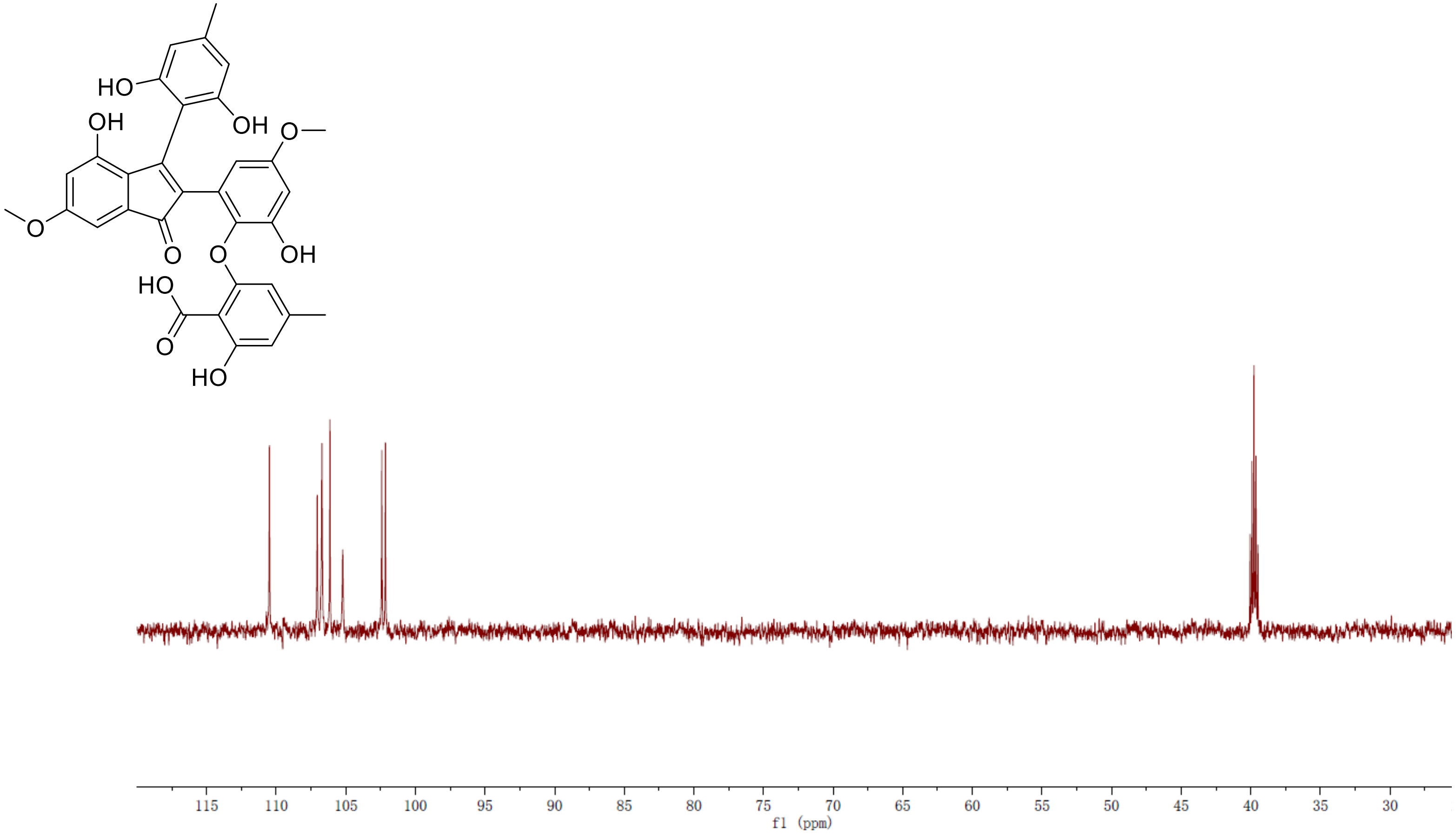

Figure S4. DEPT-135 °spectrum of compound **1** in DMSO-d_6 (150 MHz).

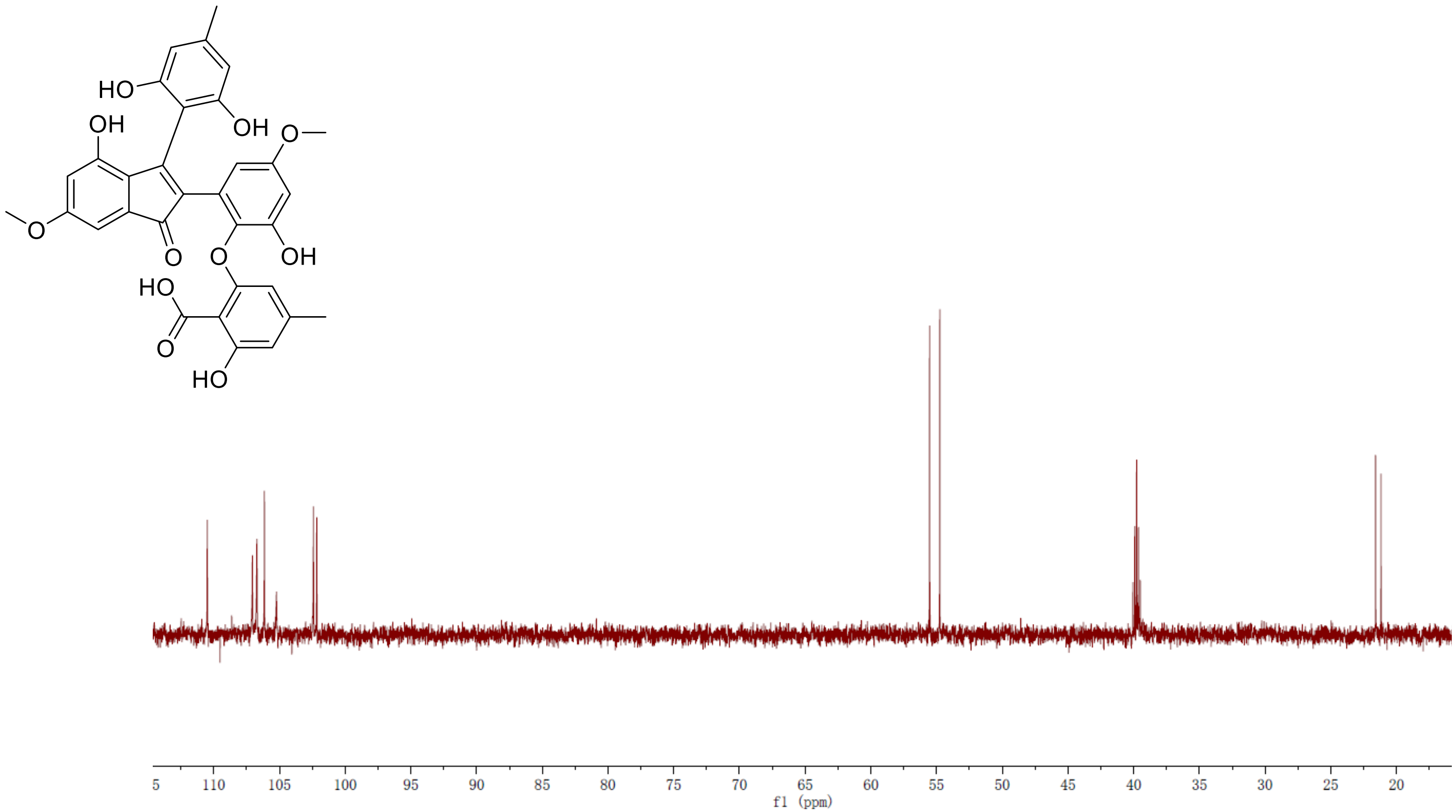

Figure S5. ^{1}H-^{1}H COSY spectrum of compound **1** in DMSO-d_6 (600 MHz).

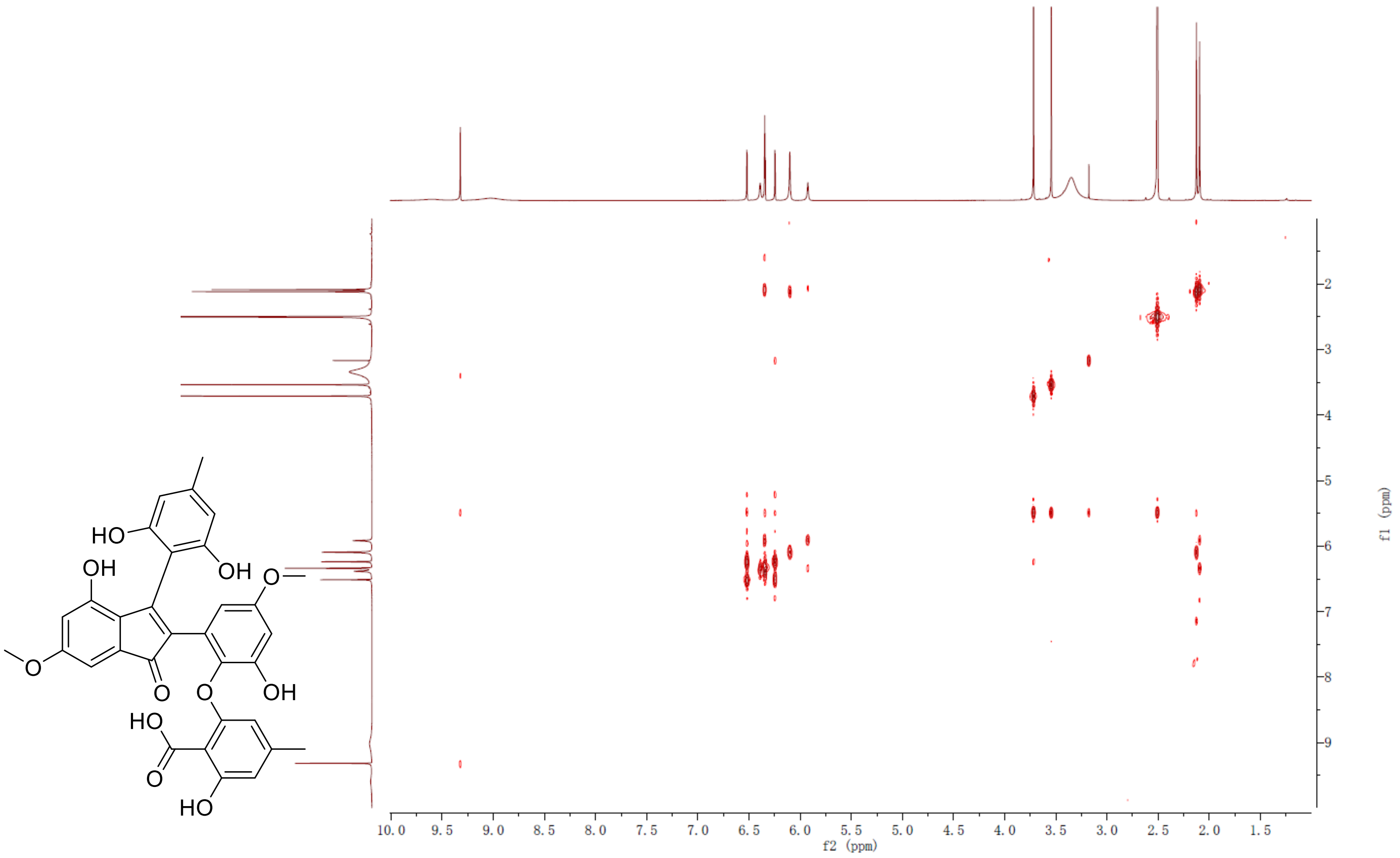

Figure S6. HSQC spectrum of compound **1** in DMSO-d_6 (600 MHz).

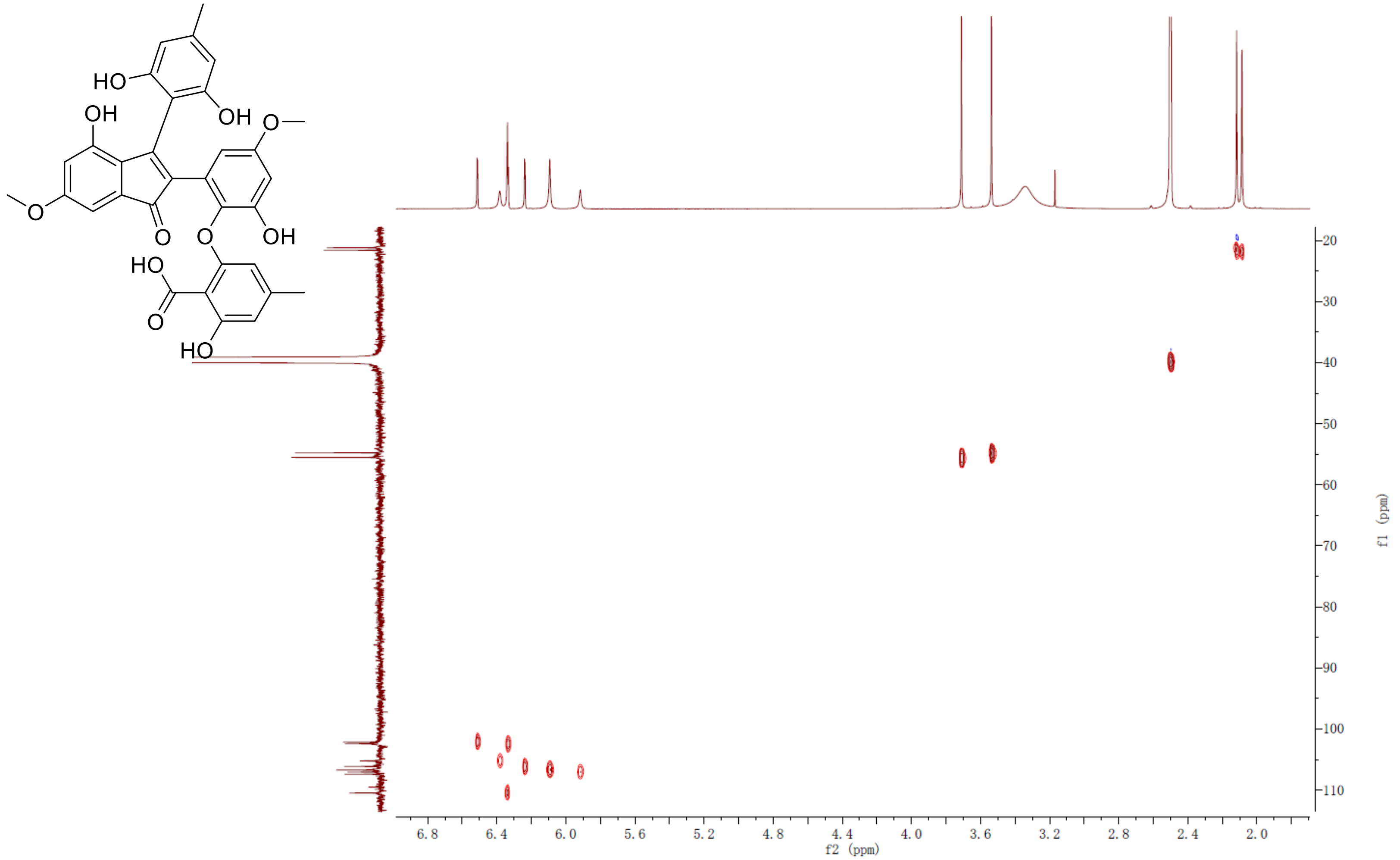

Figure S7. HMBC spectrum of compound **1** in DMSO-d_6 (600 MHz).

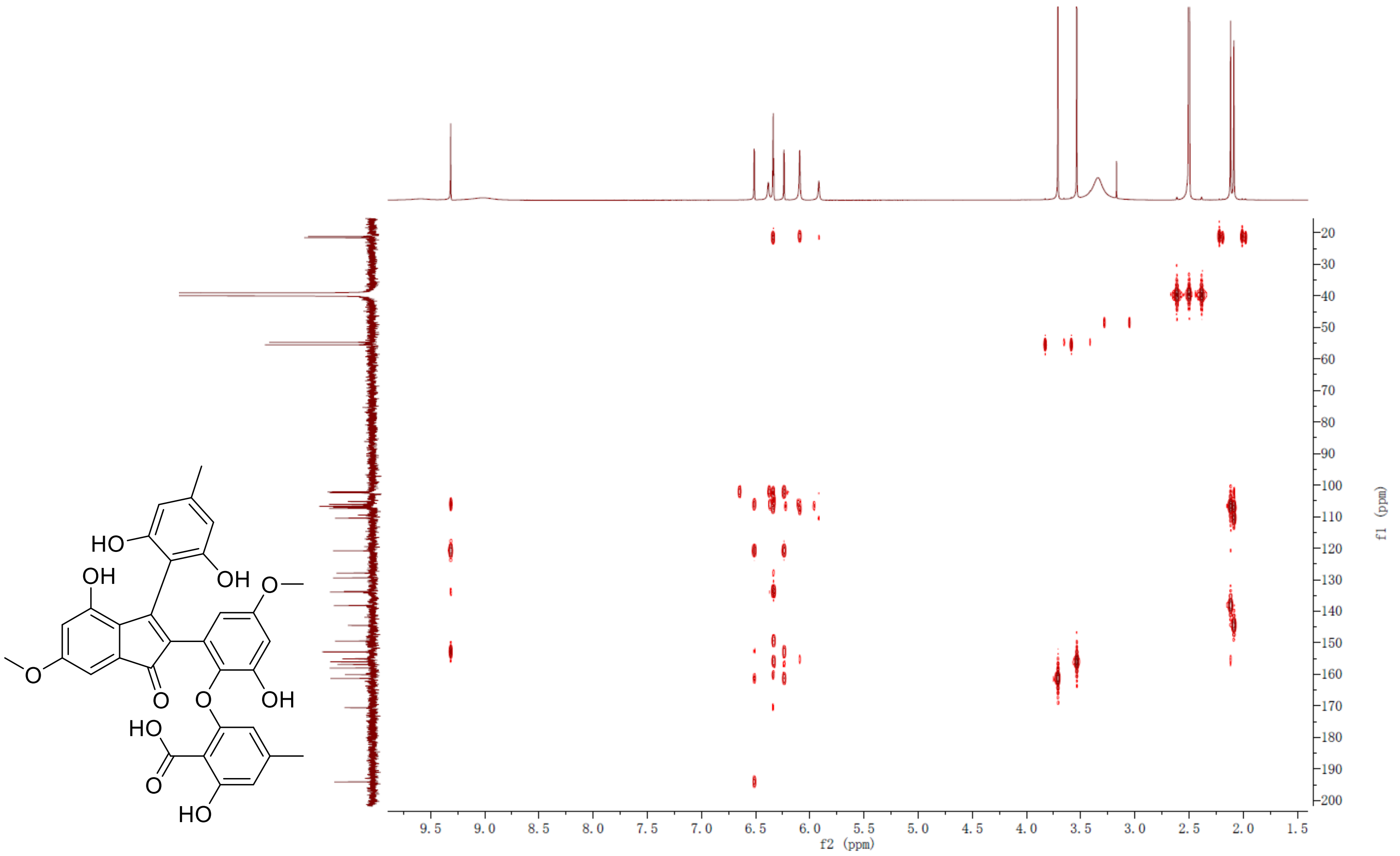

Figure S8. ^{1}H NMR spectrum of compound **2** in DMSO-d_6 (600 MHz).

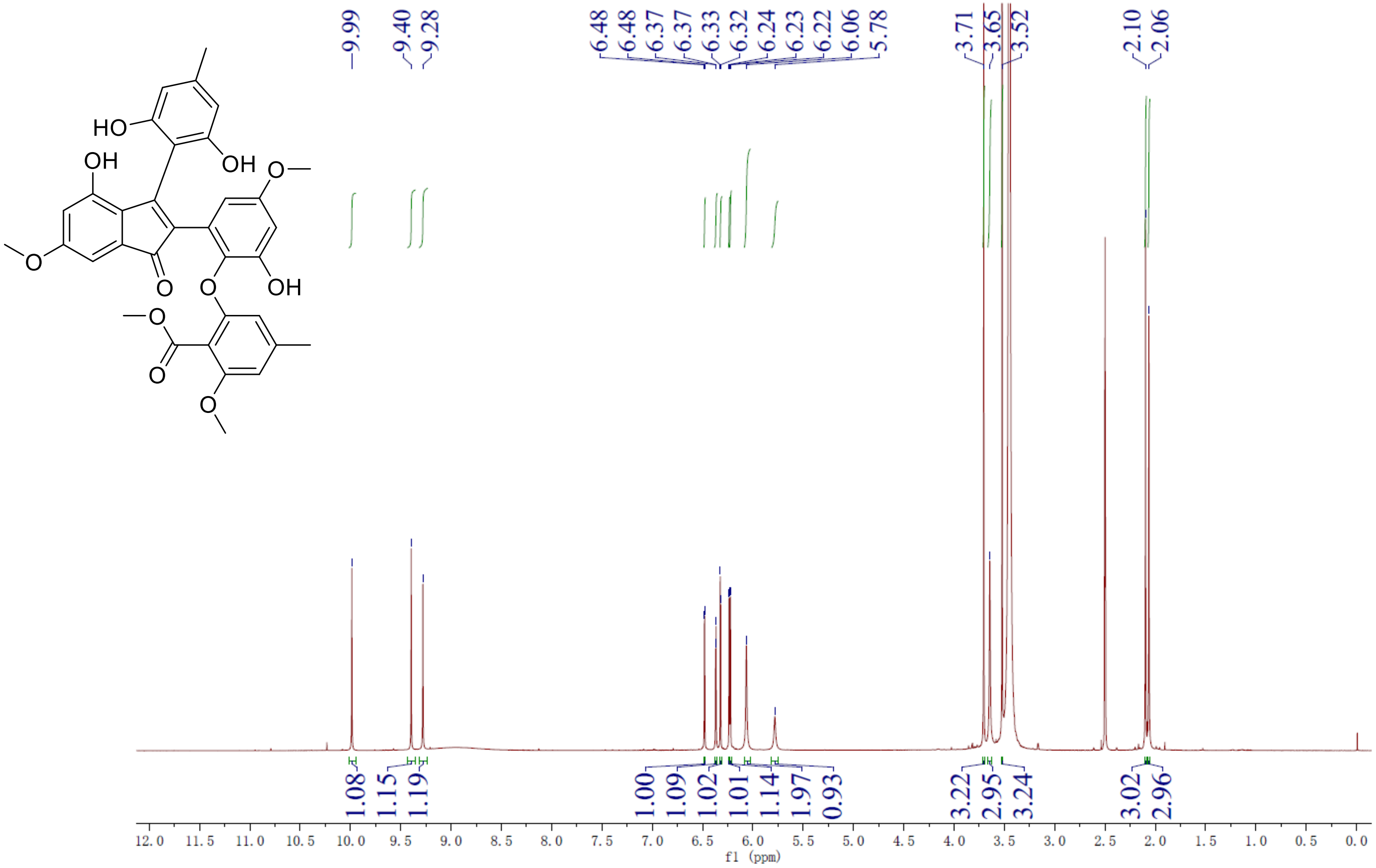

Figure S9. ^{13}C NMR spectrum of compound **2** in DMSO-d_6 (150 MHz).

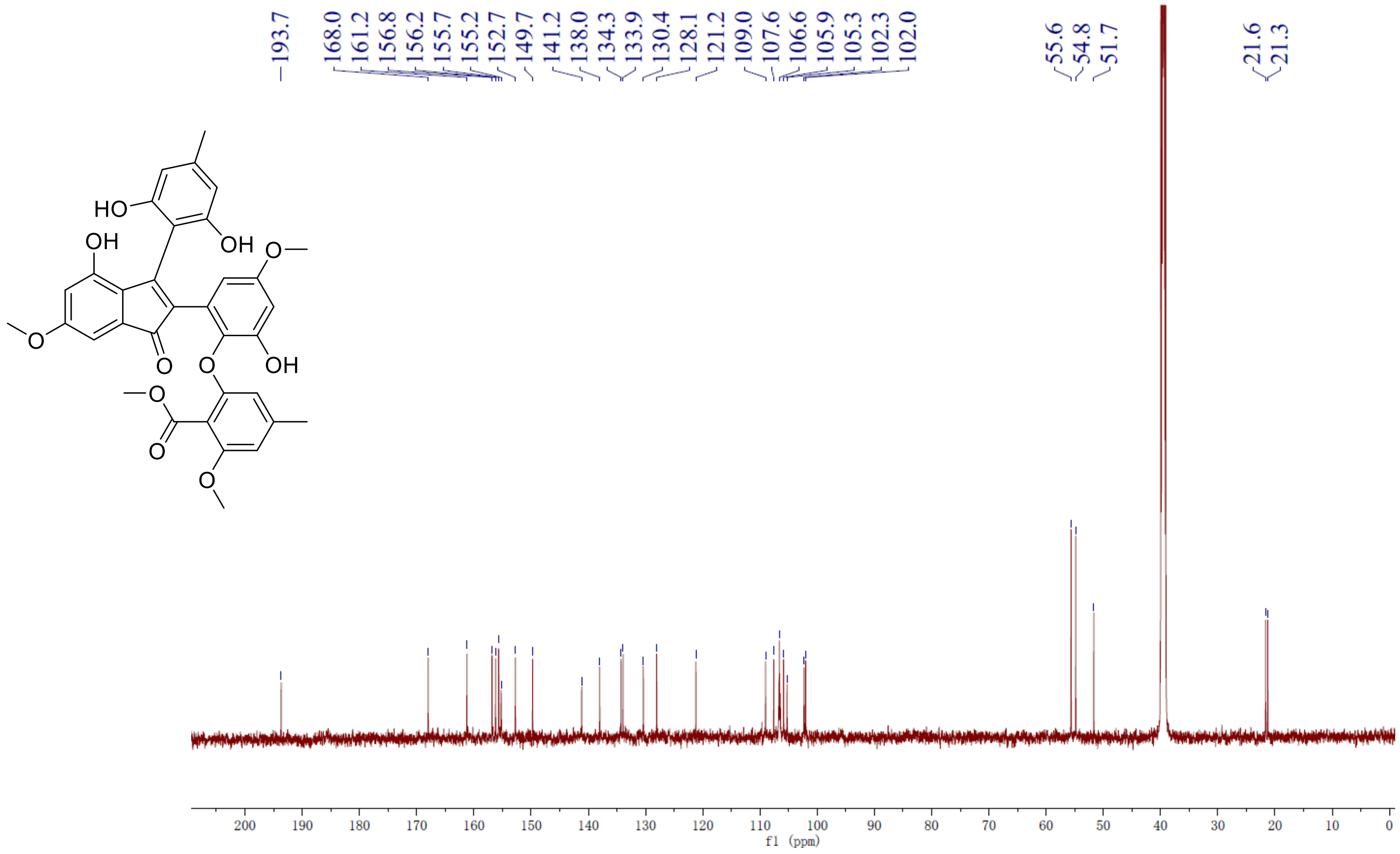

Figure S10. DEPT-90 °spectrum of compound **2** in DMSO-d_6 (150 MHz).

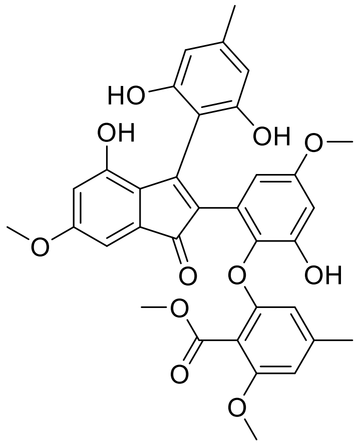

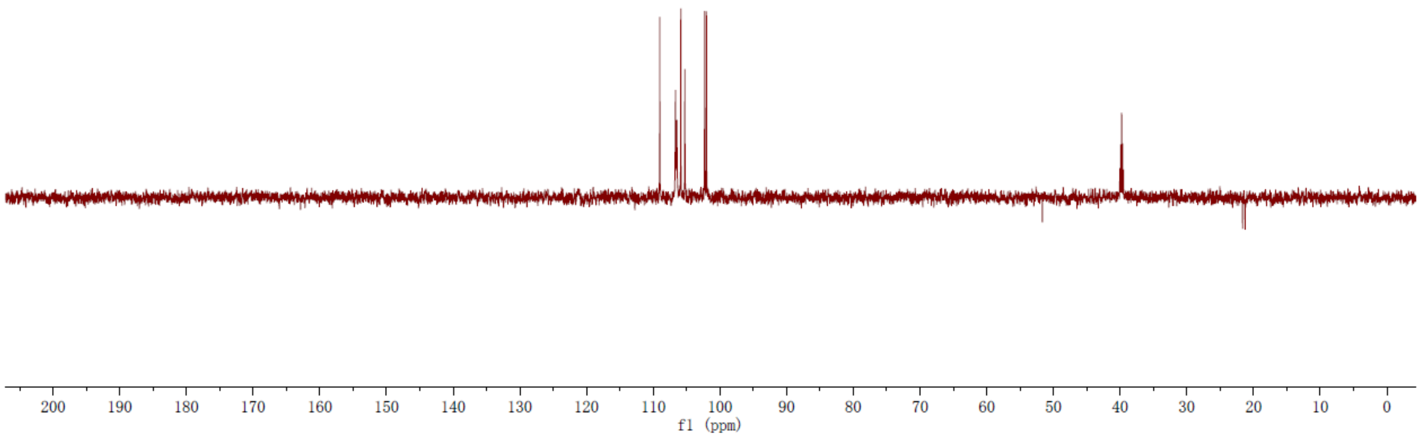

Figure S11. DEPT-135 °spectrum of compound **2** in DMSO-d_6 (150 MHz).

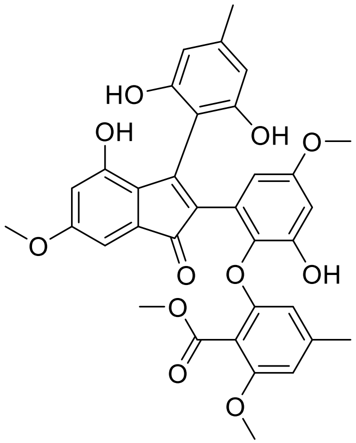

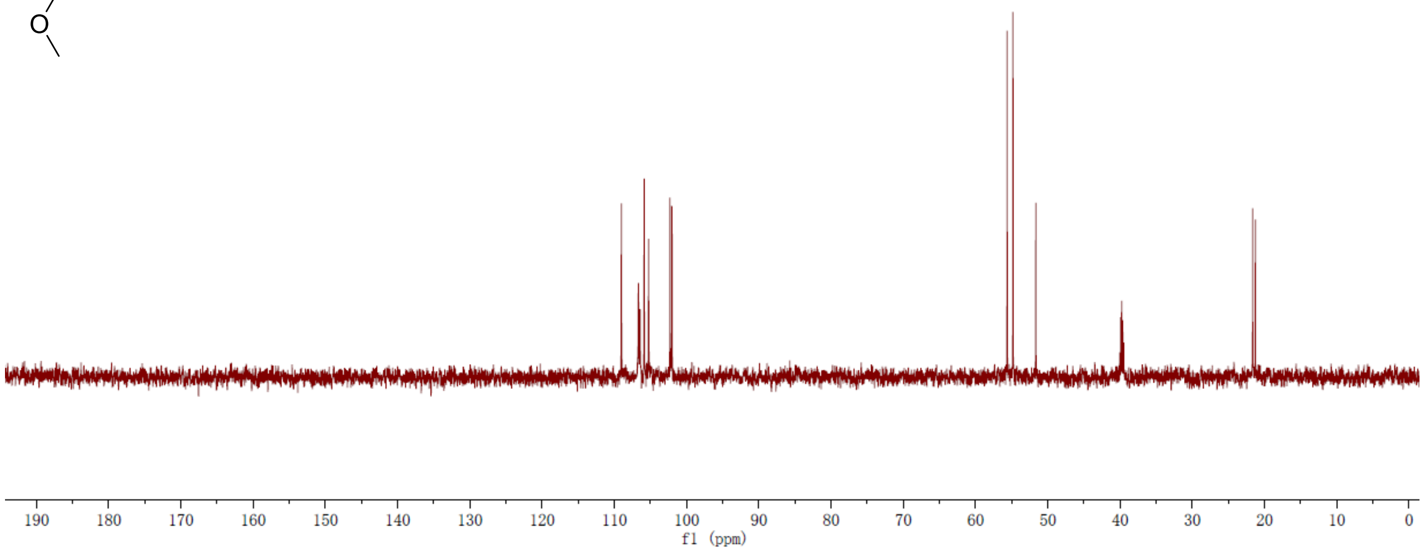

Figure S12. ^{1}H-^{1}H COSY spectrum of compound **2** in DMSO-d_6 (600 MHz).

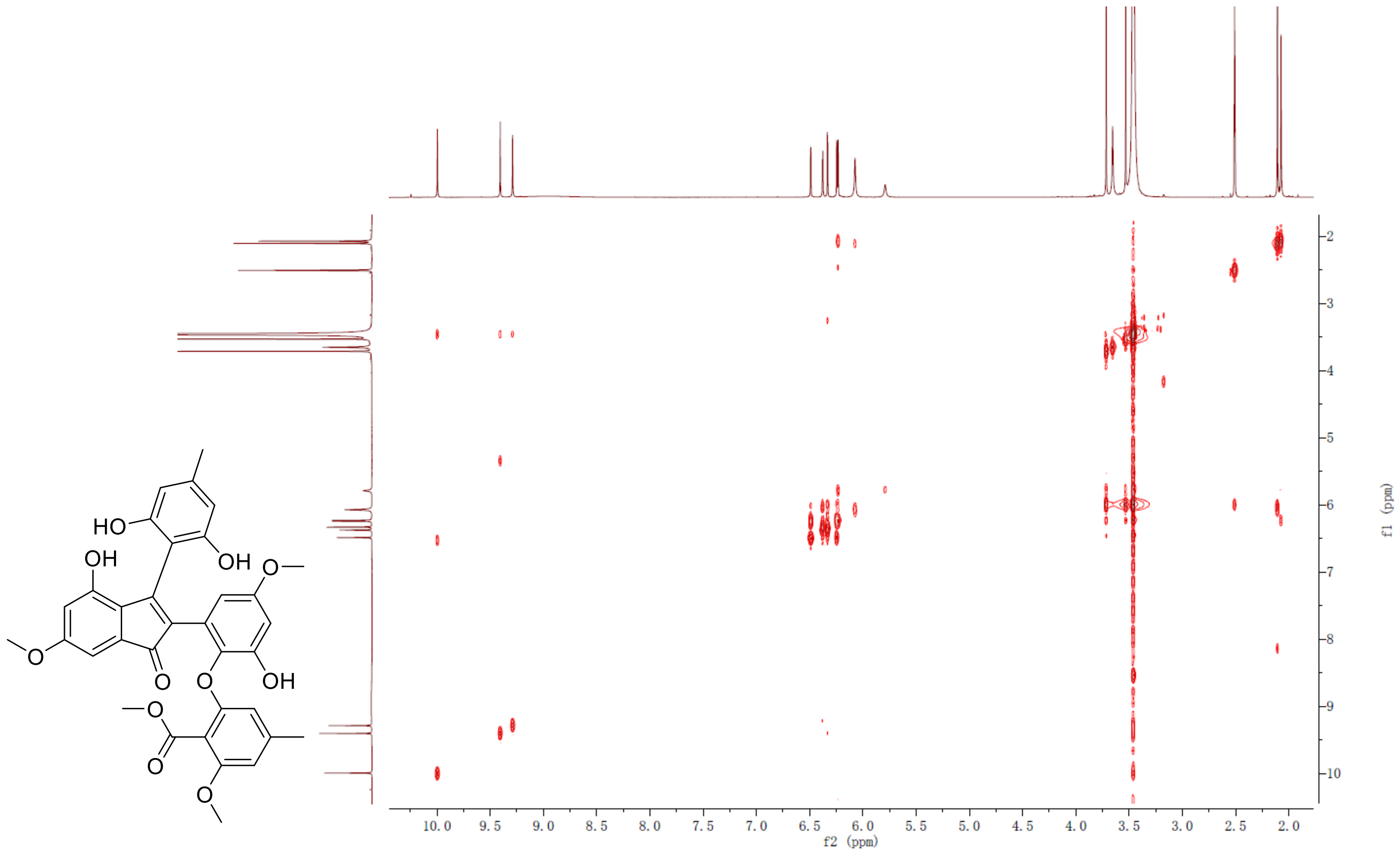

Figure S13. HSQC spectrum of compound **2** in DMSO-d_6 (600 MHz).

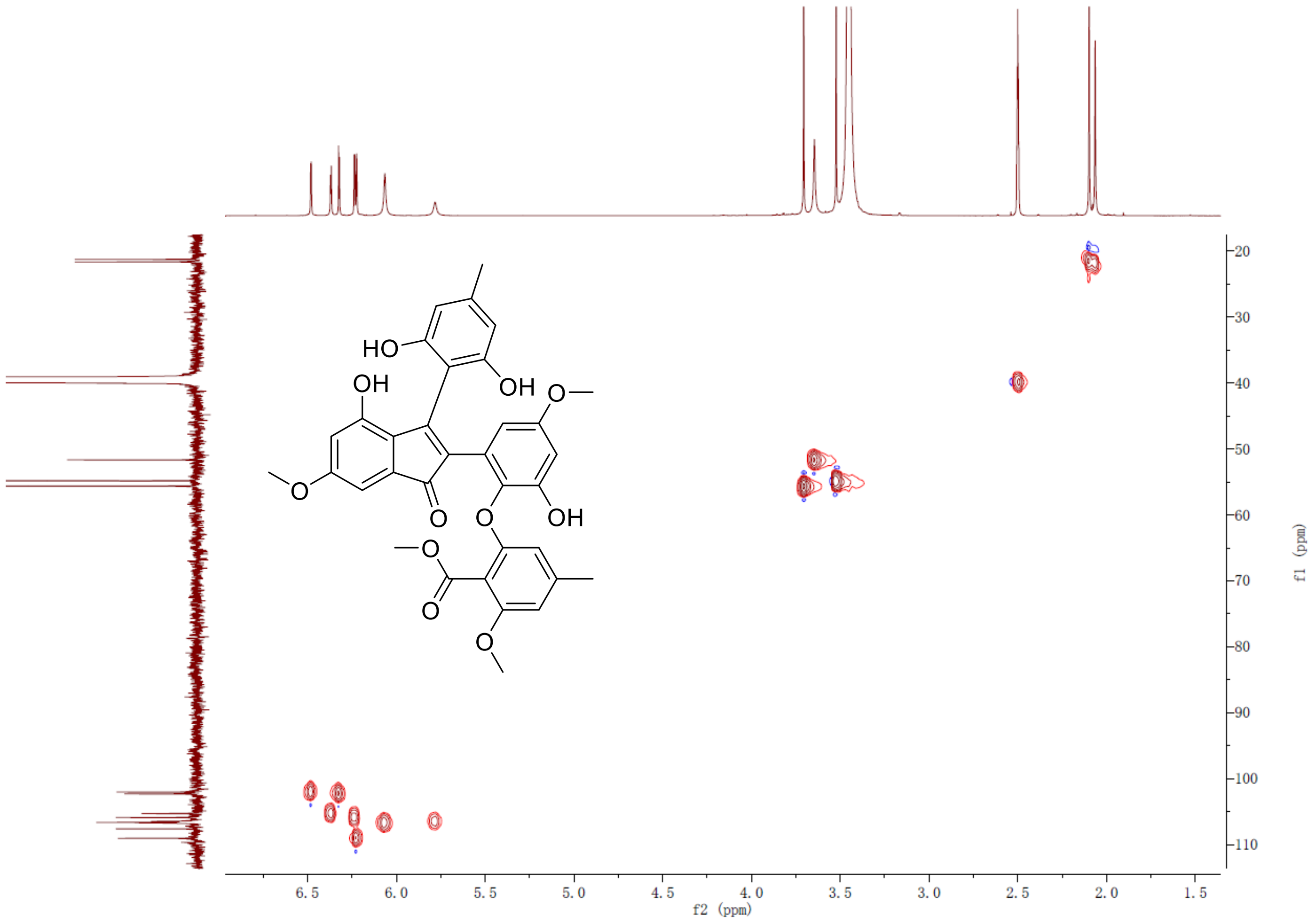

Figure S14. HMBC spectrum of compound **2** in DMSO-d_6 (600 MHz).

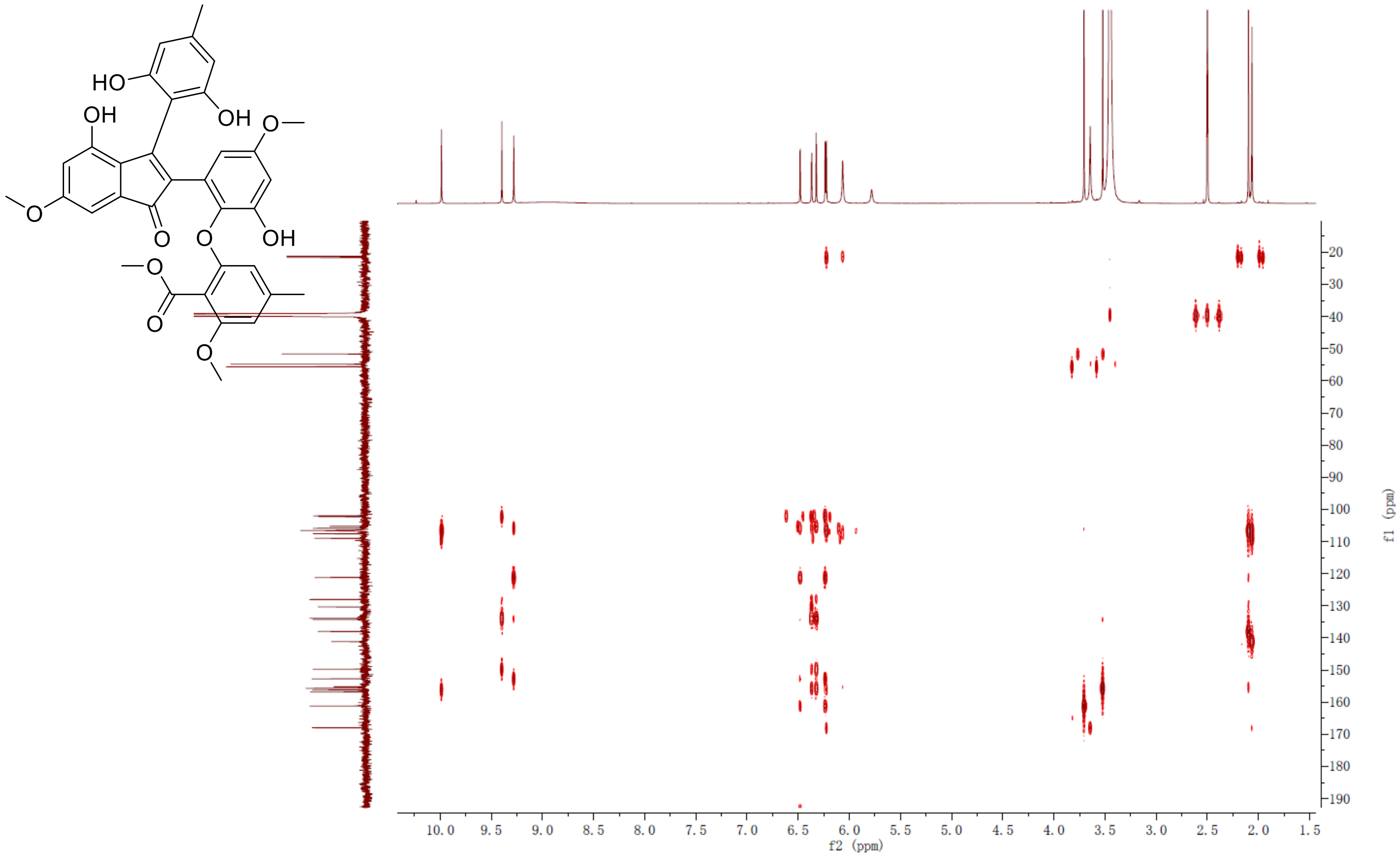

Figure S15. ^{1}H NMR spectrum of compound **3** in acetone-d_6 (600 MHz).

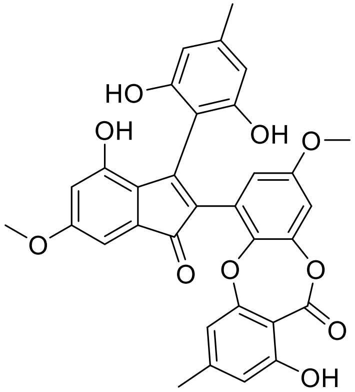

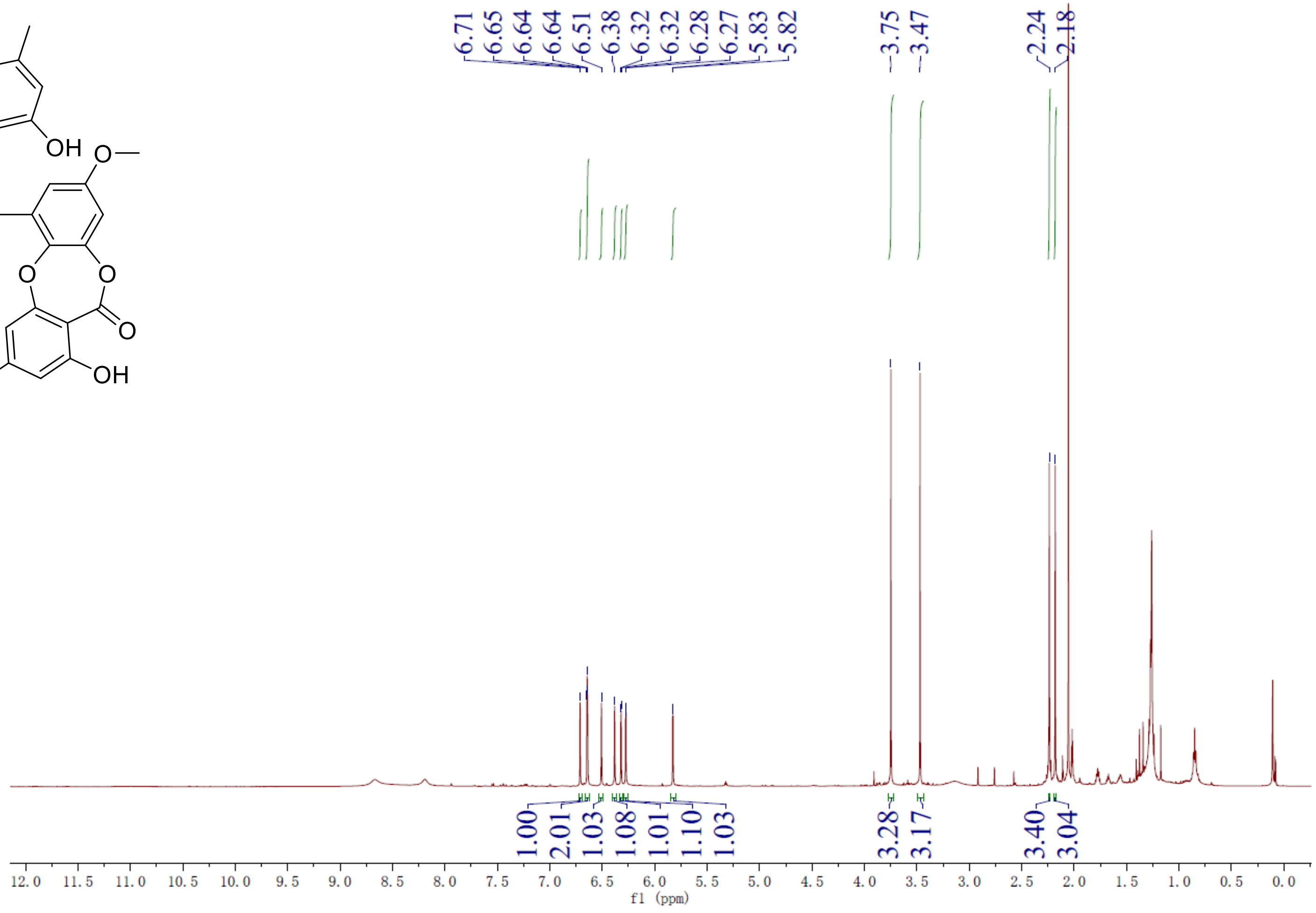

Figure S16. ^{13}C NMR spectrum of compound **3** in acetone-d_6 (150 MHz).

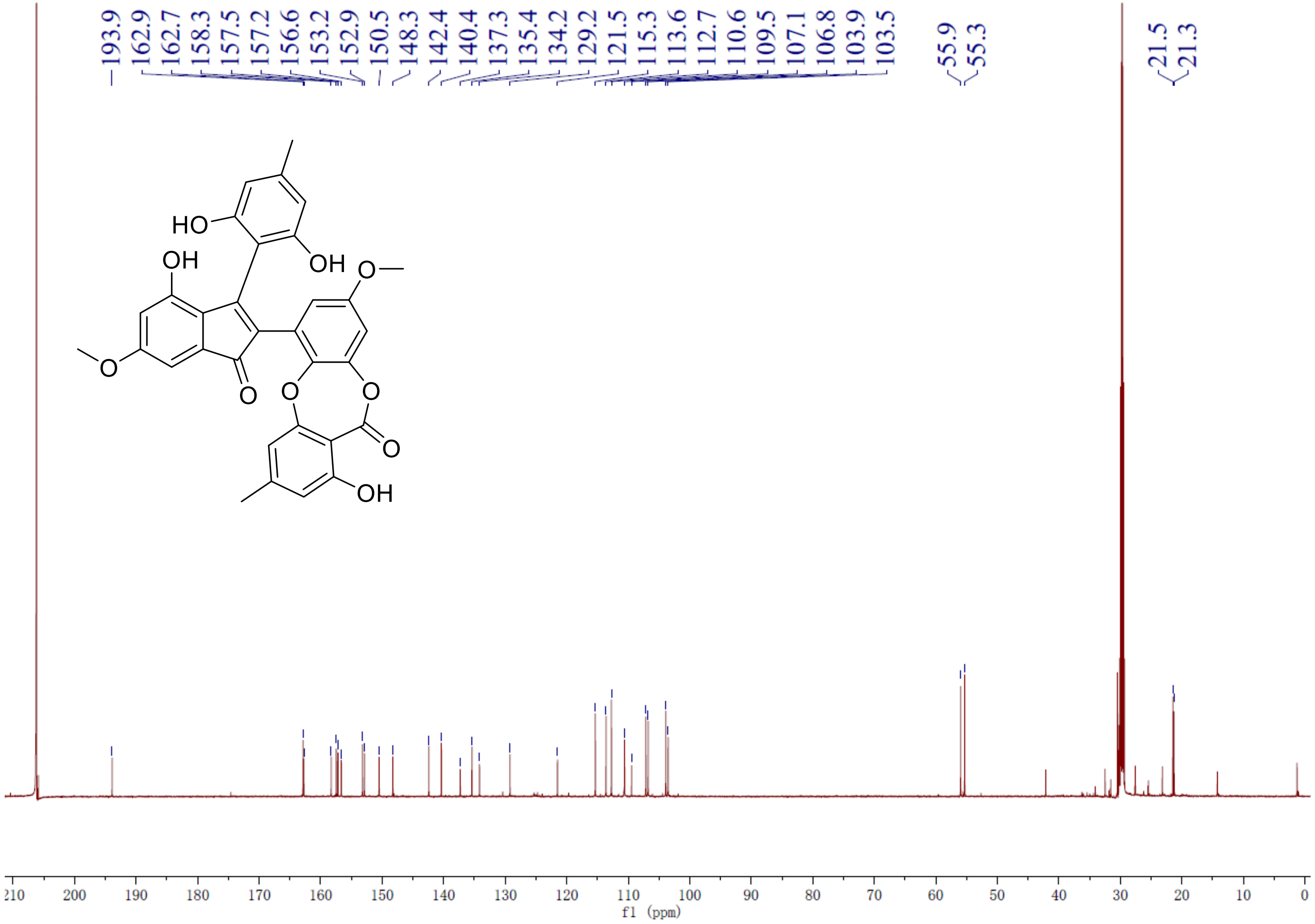

Figure S17. DEPT-90 °spectrum of compound **3** in acetone-d_6 (150 MHz).

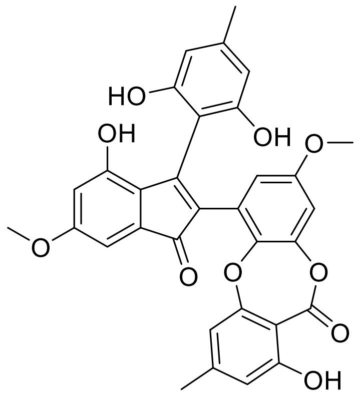

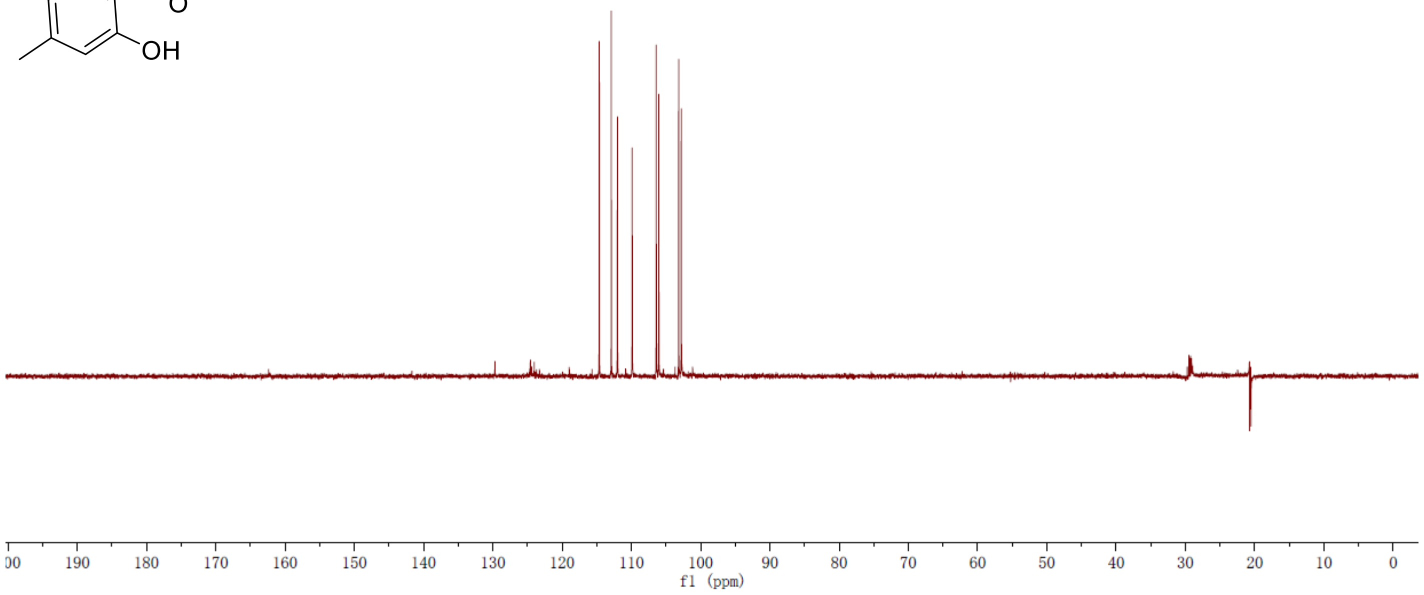

Figure S18. DEPT-135 °spectrum of compound **3** in acetone-d_6 (150 MHz).

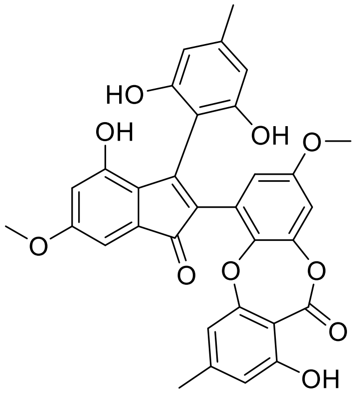

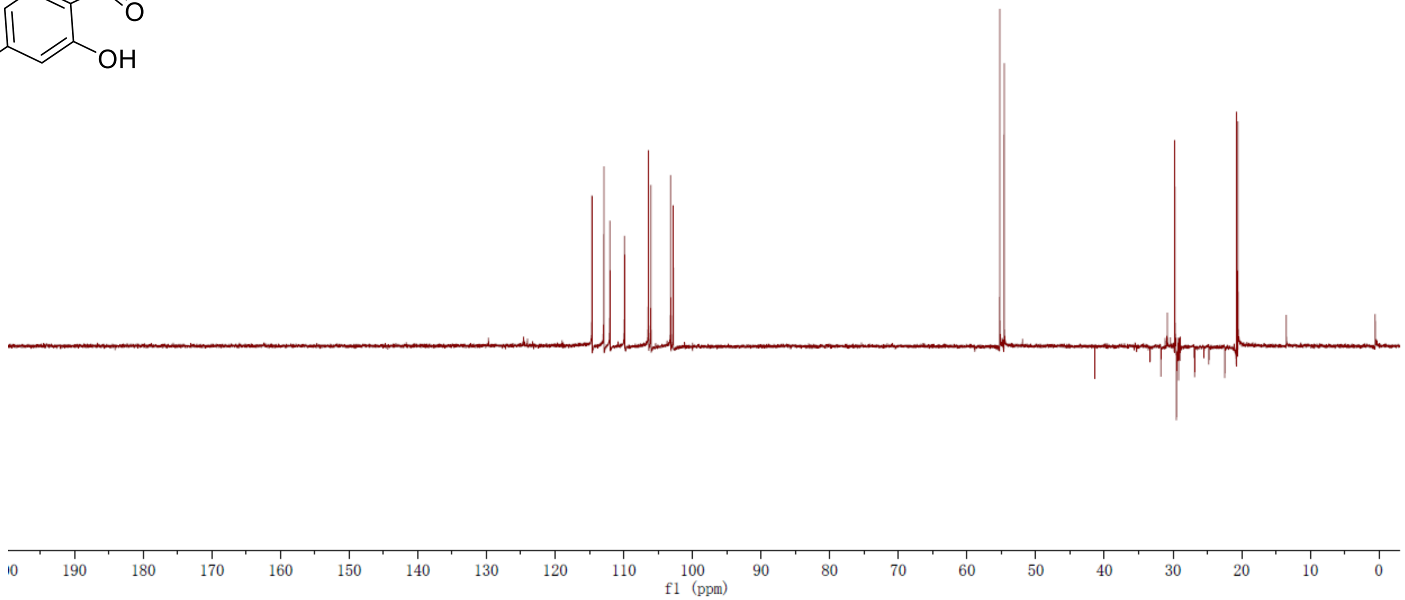

Figure S19. ^{1}H-^{1}H COSY spectrum of compound **3** in acetone-d_6 (600 MHz).

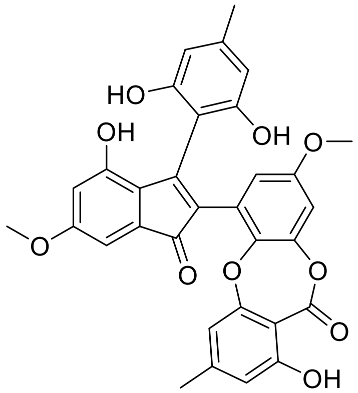

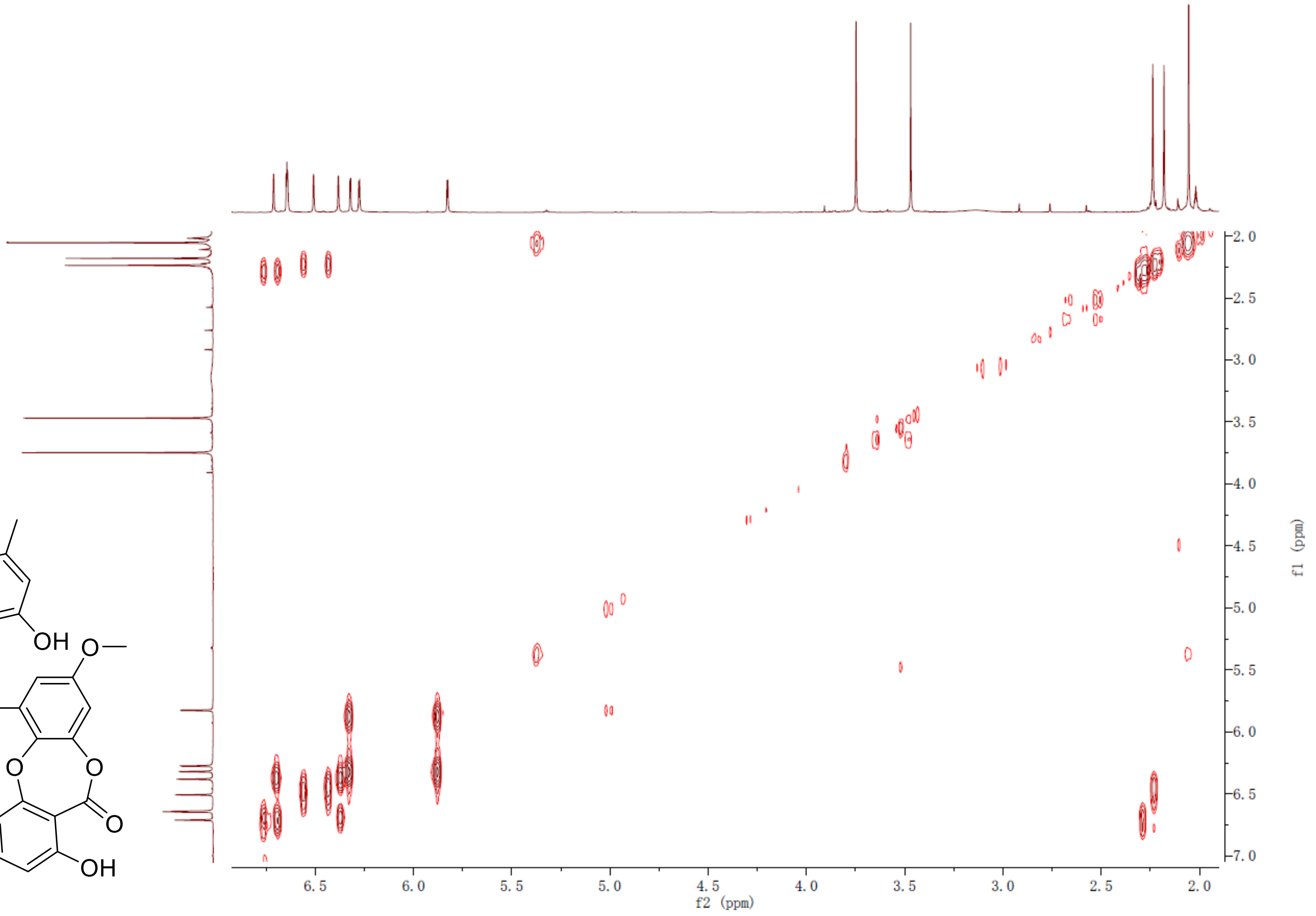

Figure S20. HSQC spectrum of compound **3** in acetone-d_6 (600 MHz).

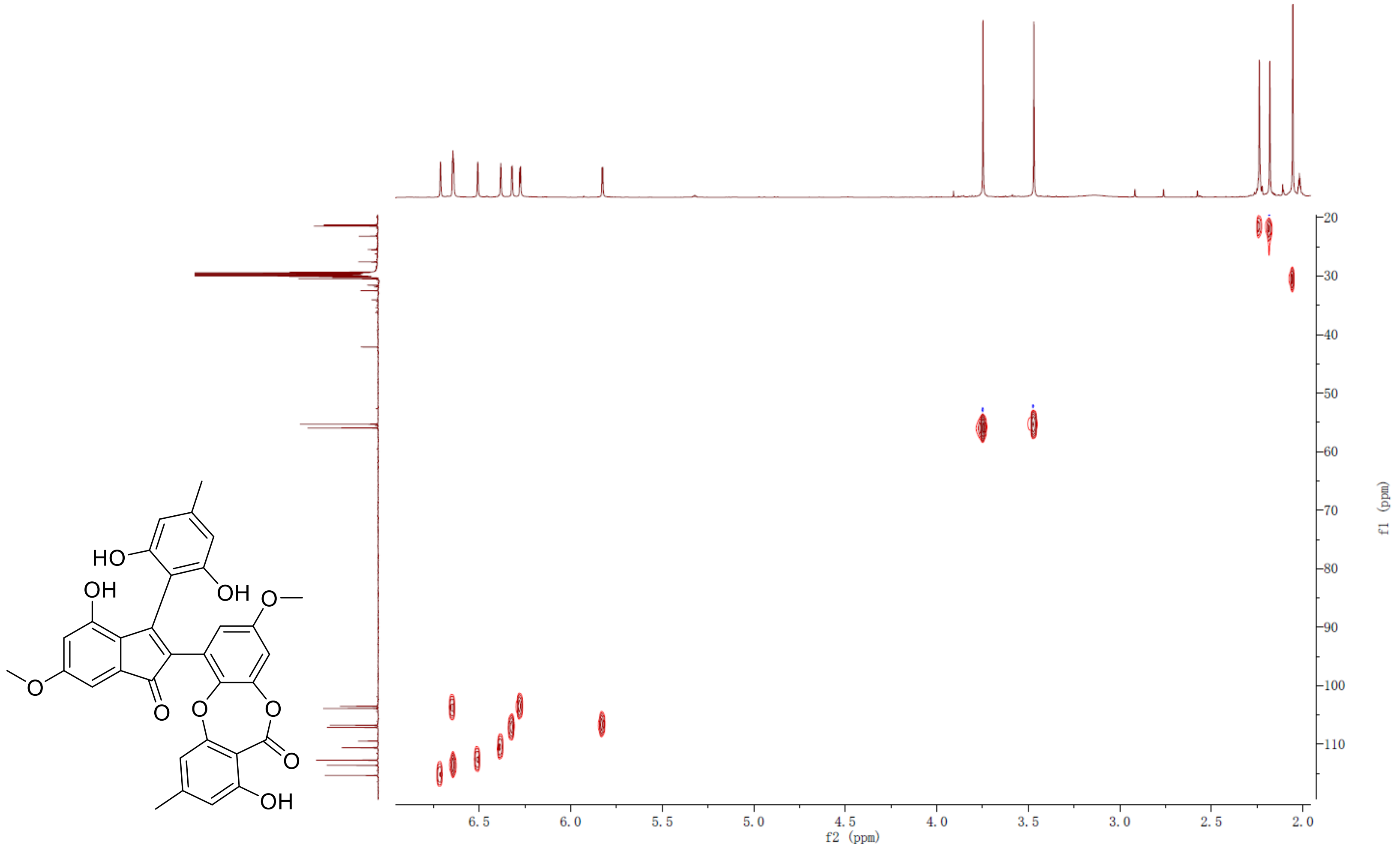

Figure S21. HSQC spectrum of compound **3** in acetone-d_6 (600 MHz).

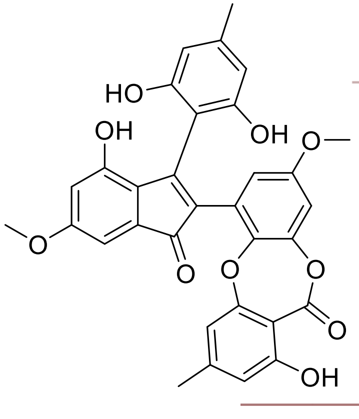

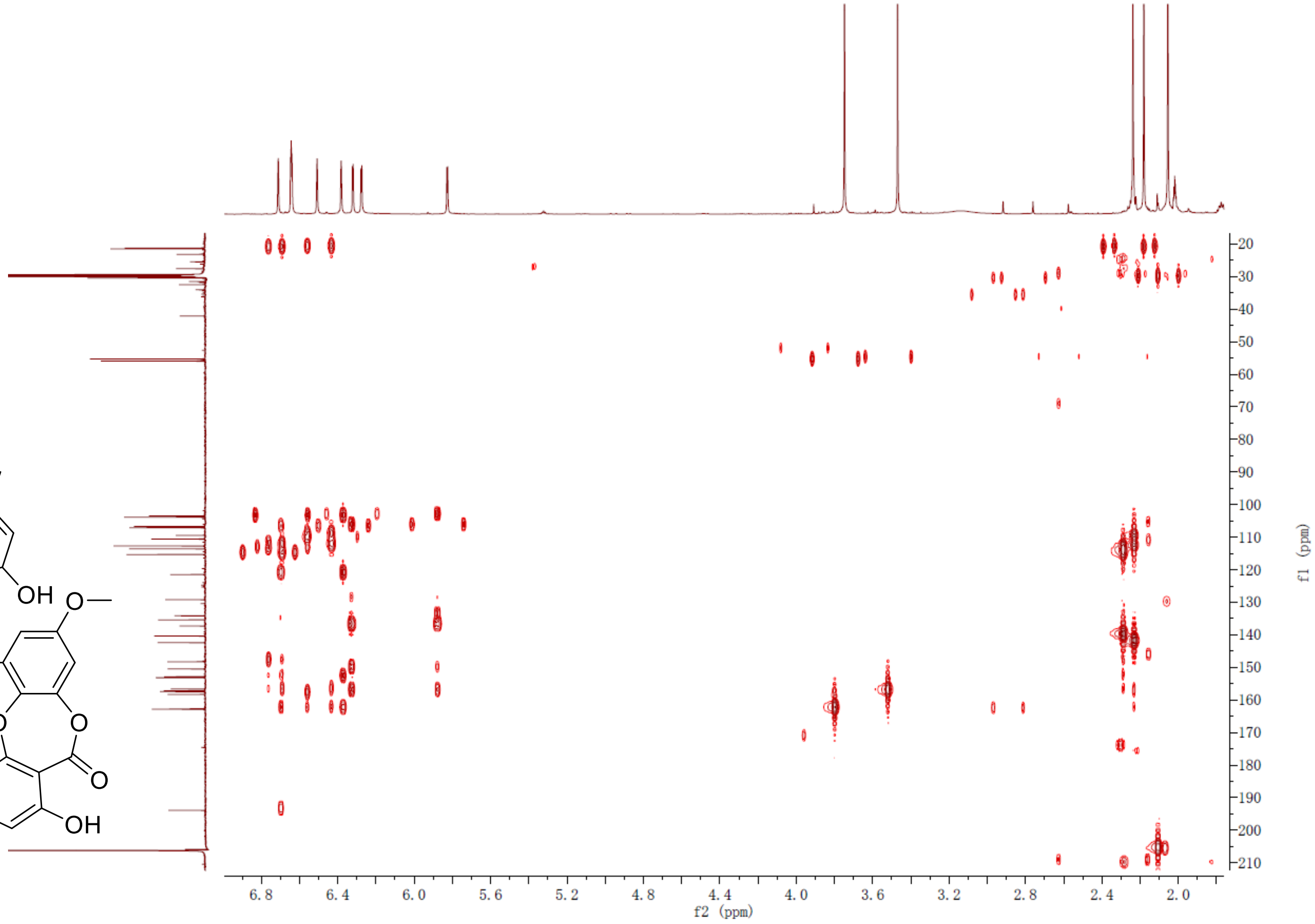

Figure S22. ^{1}H NMR spectrum of compound **4** in mathanol-d_4 (500 MHz).

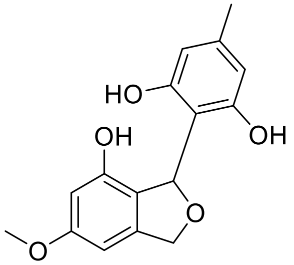

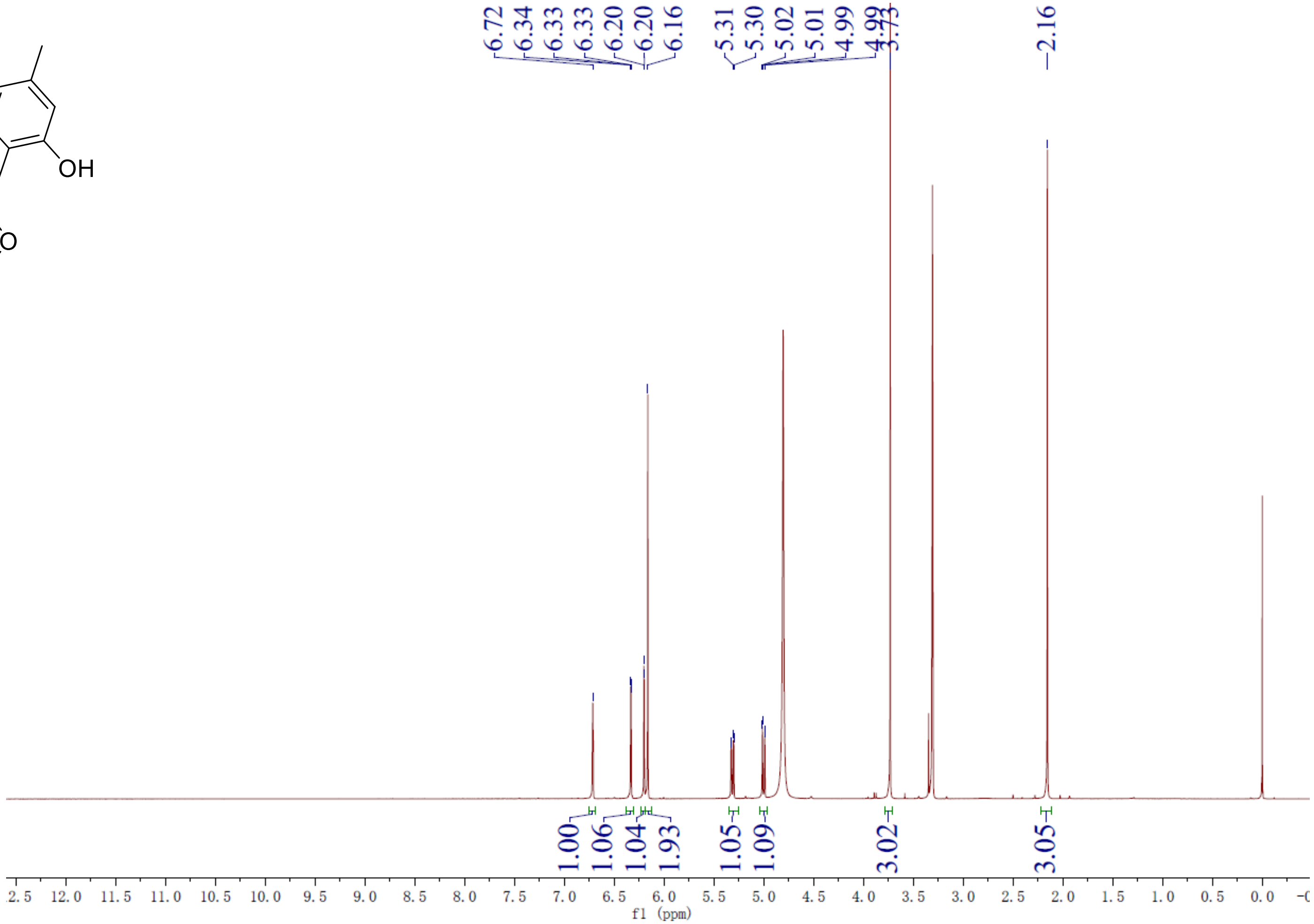

Figure S23. ^{13}C NMR spectrum of compound **4** in mathanol-d_4 (125 MHz).

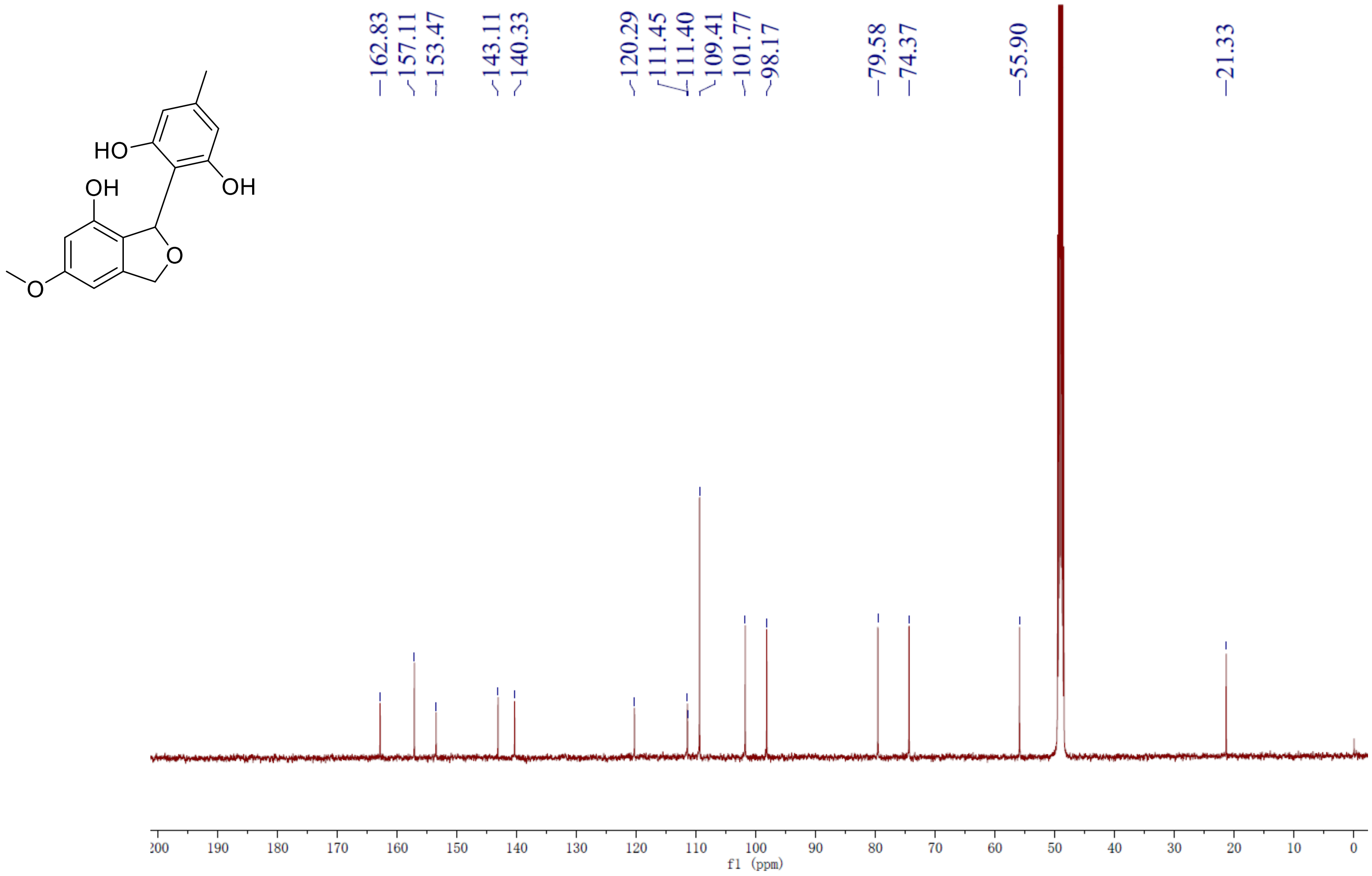

Figure S24. DEPT-90 °spectrum of compound **4** in mathanol-d_4 (125 MHz).

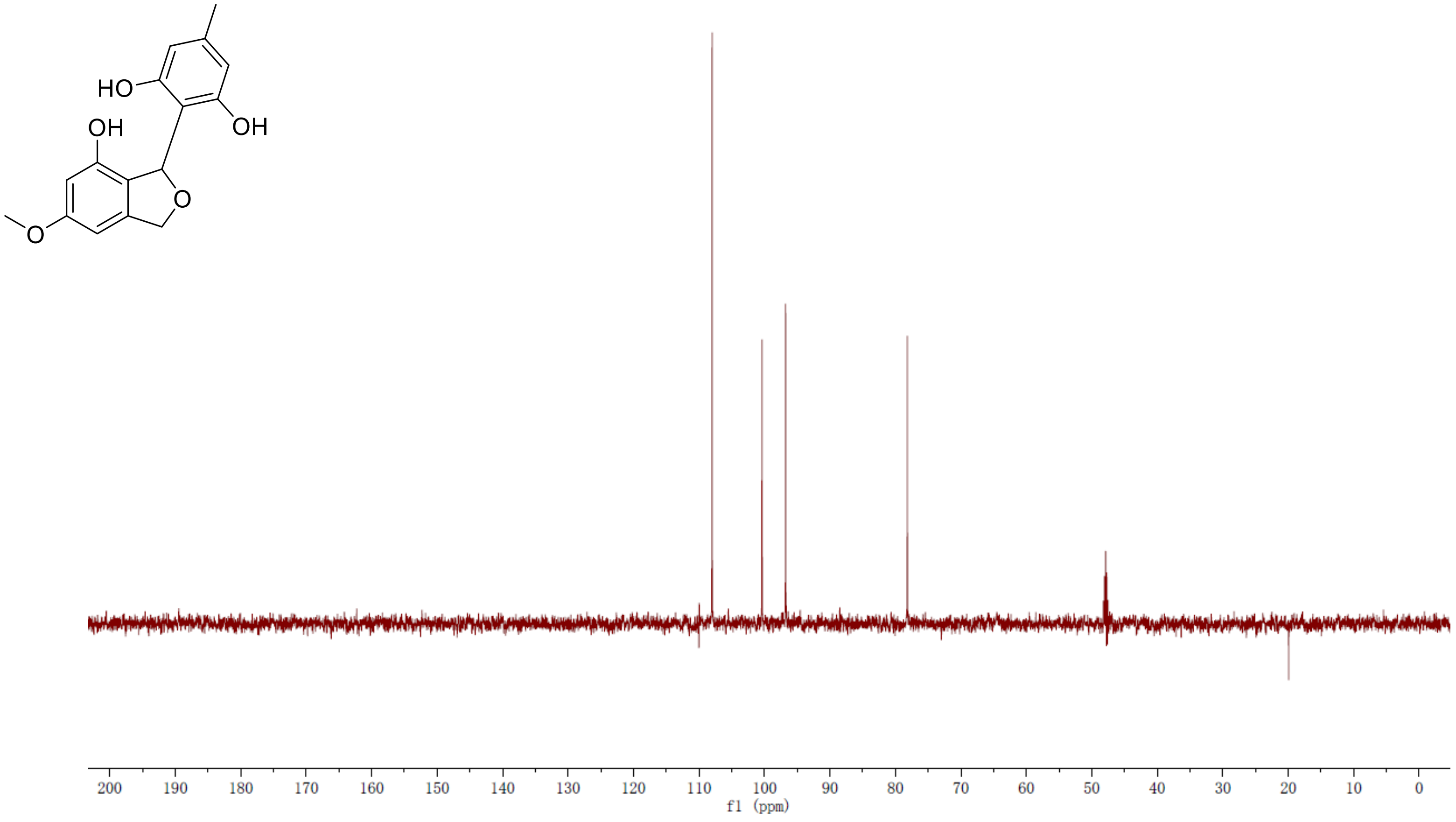

Figure S25. DEPT-135 °spectrum of compound **4** in mathanol-d_4 (125 MHz).

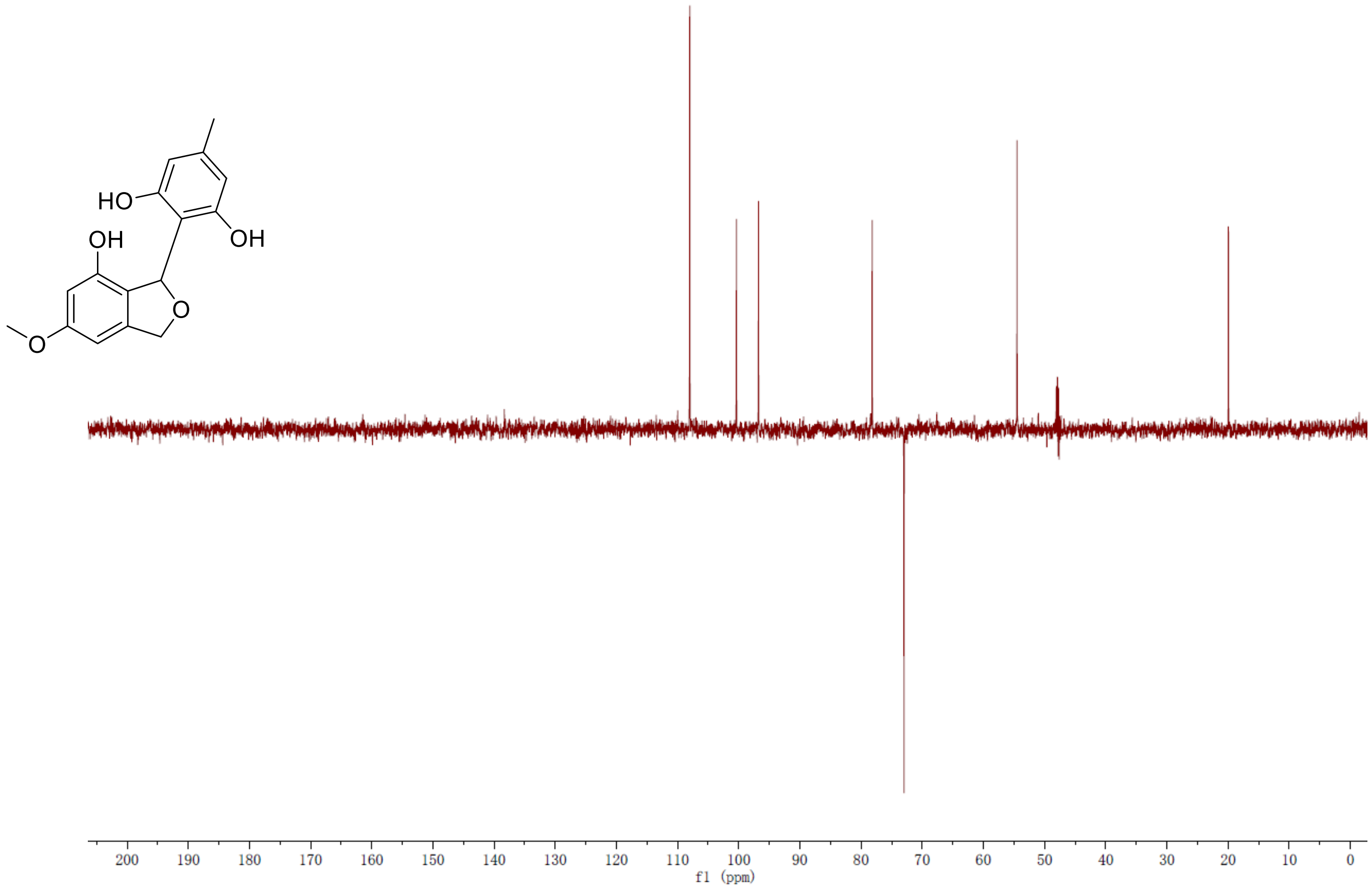

Figure S26. ^{1}H-^{1}H COSY spectrum of compound **4** in methanol-d_4 (600 MHz).

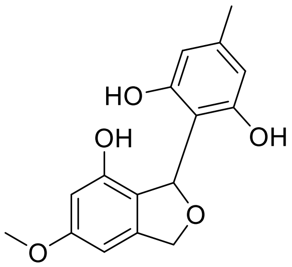

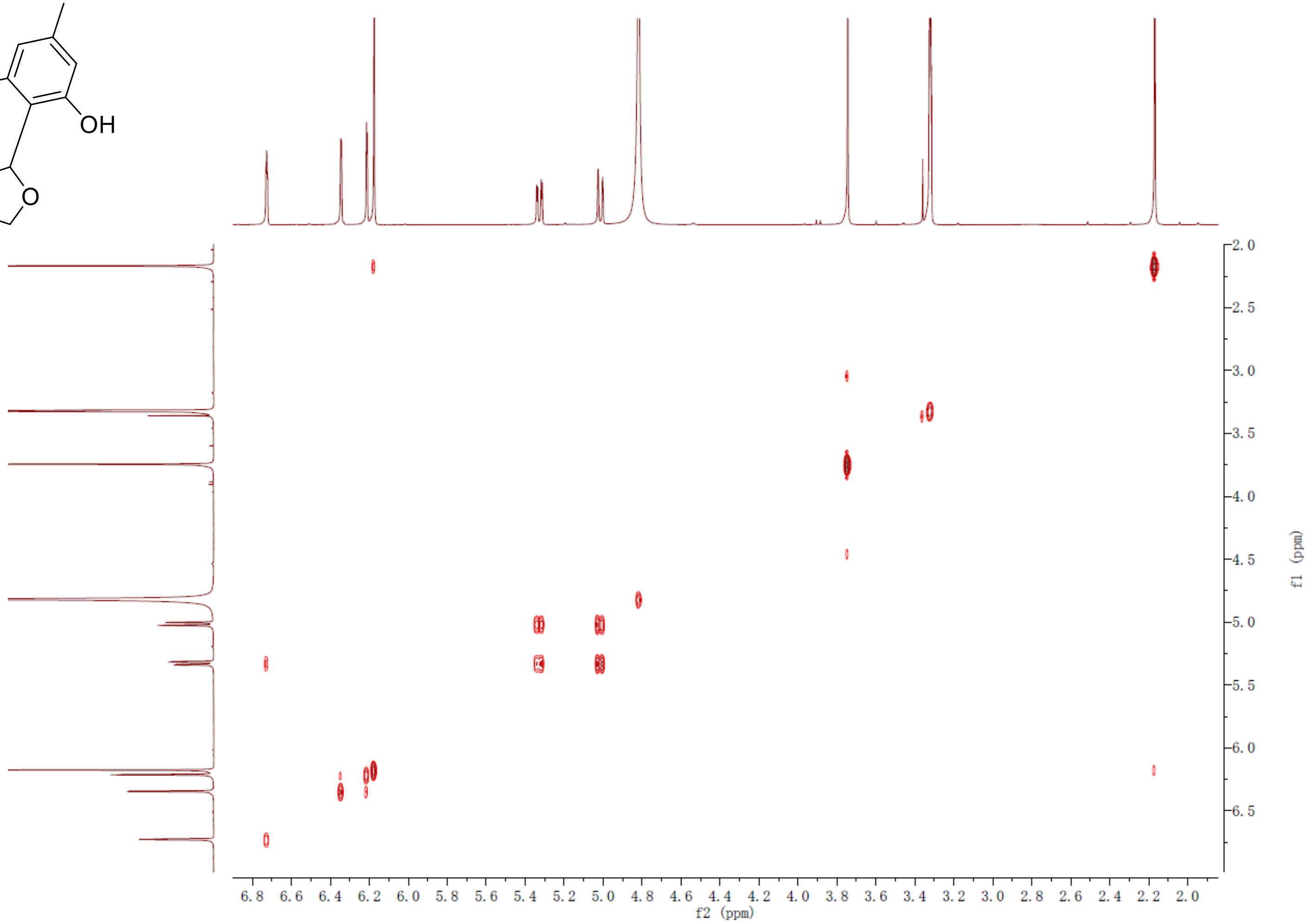

Figure S27. HSQC spectrum of compound **4** in methanol-d_4 (600 MHz).

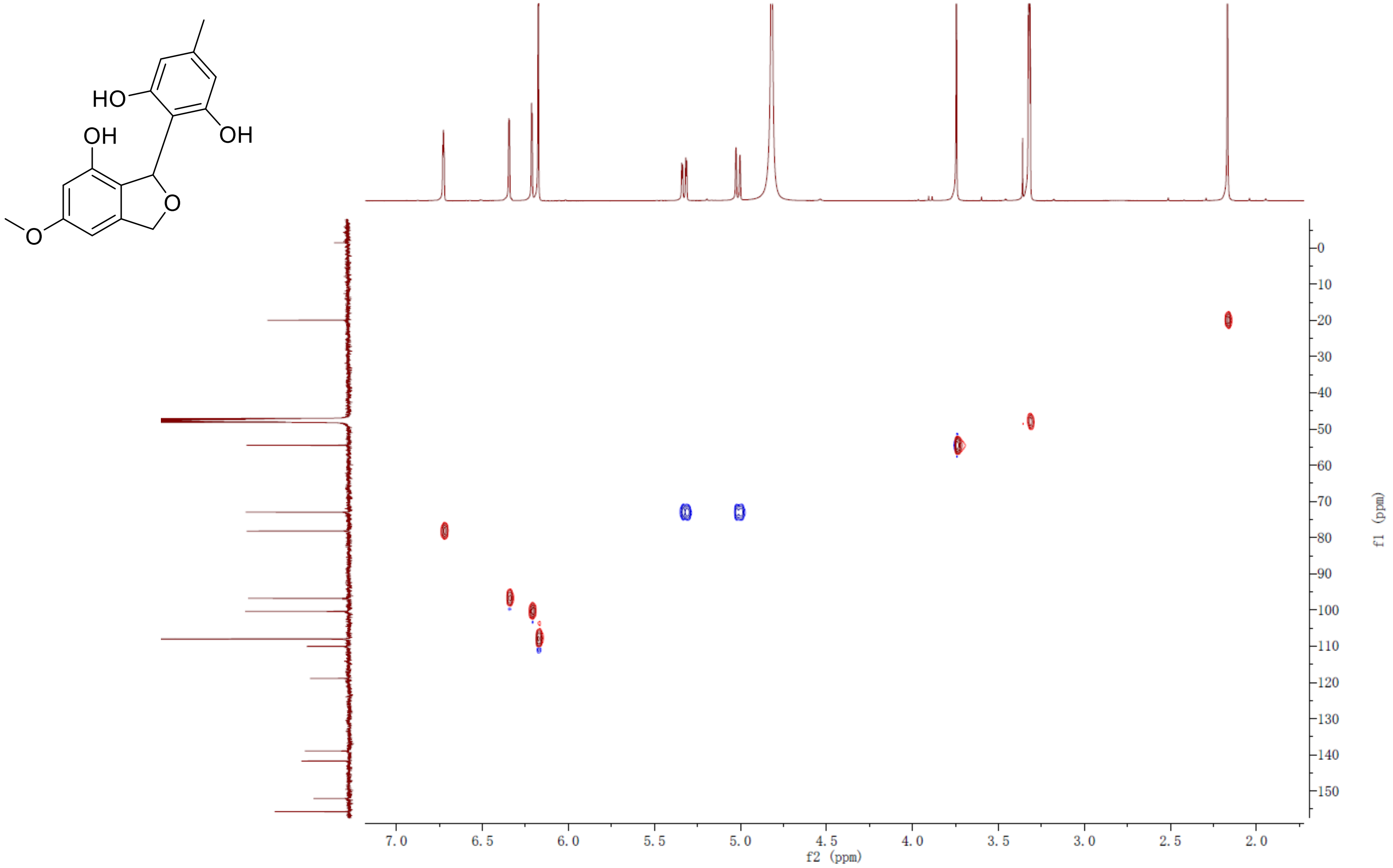

Figure S28. HMBC spectrum of compound **4** in methanol-d_4 (600 MHz).

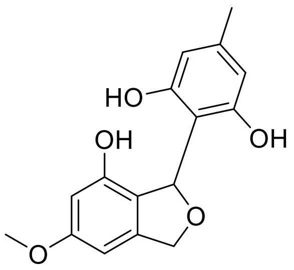

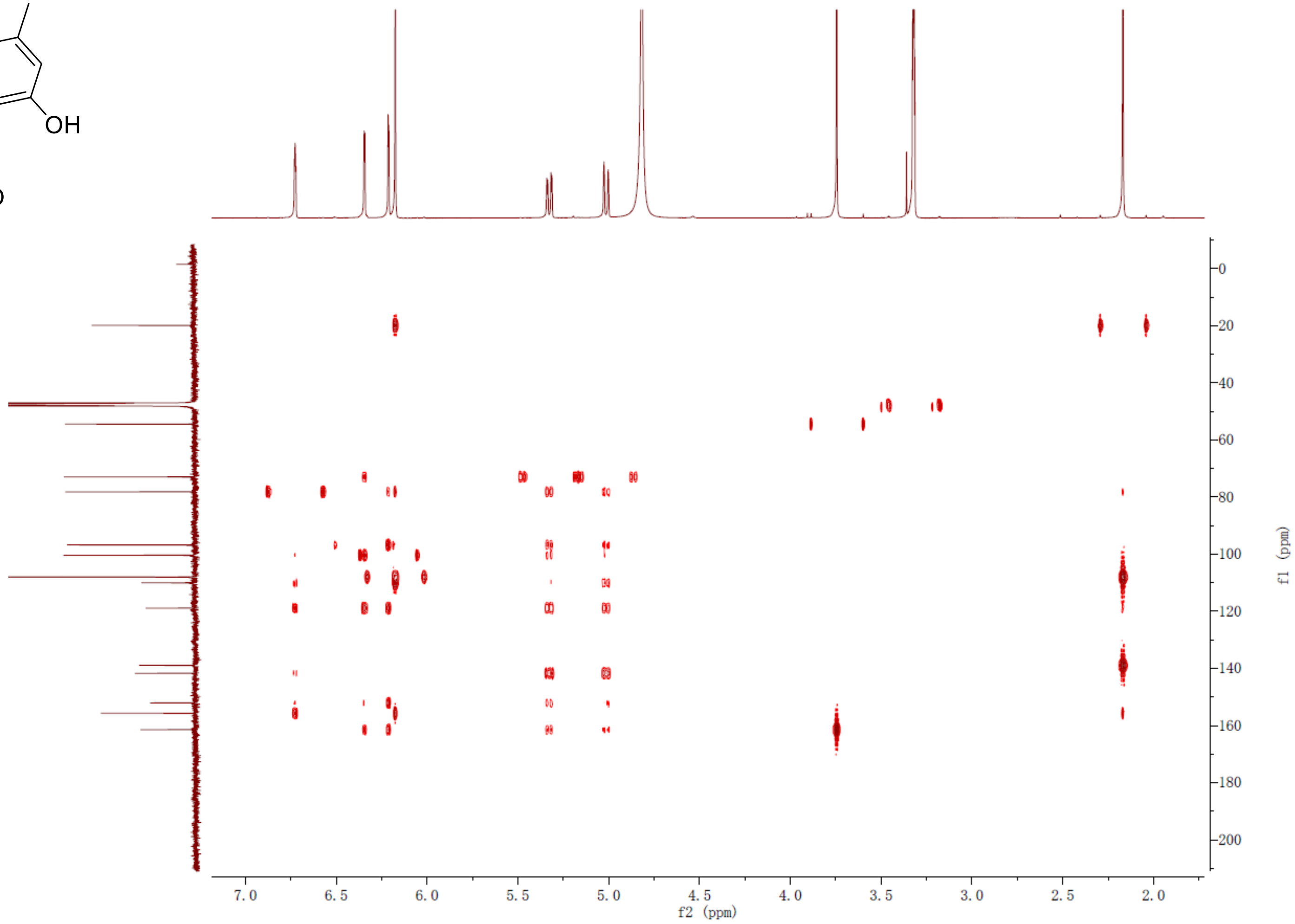

Figure S29. ^{1}H NMR spectrum of compound **5** in methanol-d_4 (500 MHz).

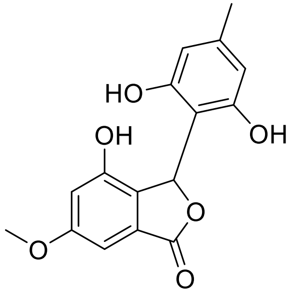

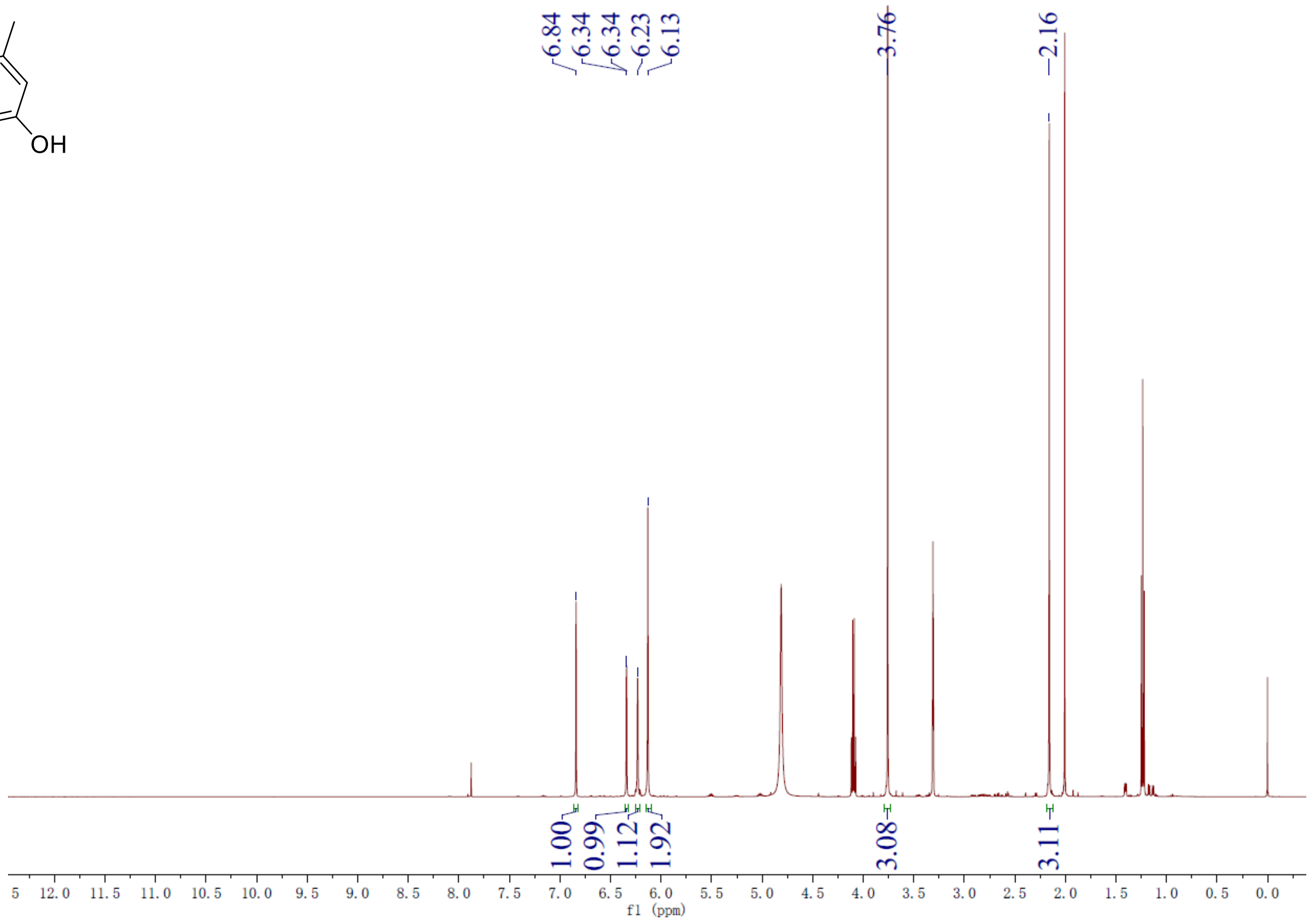

Figure S30. ^{13}C NMR spectrum of compound **5** in mathanol-d_4 (125 MHz).

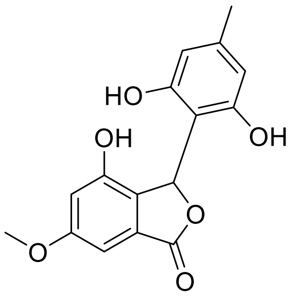

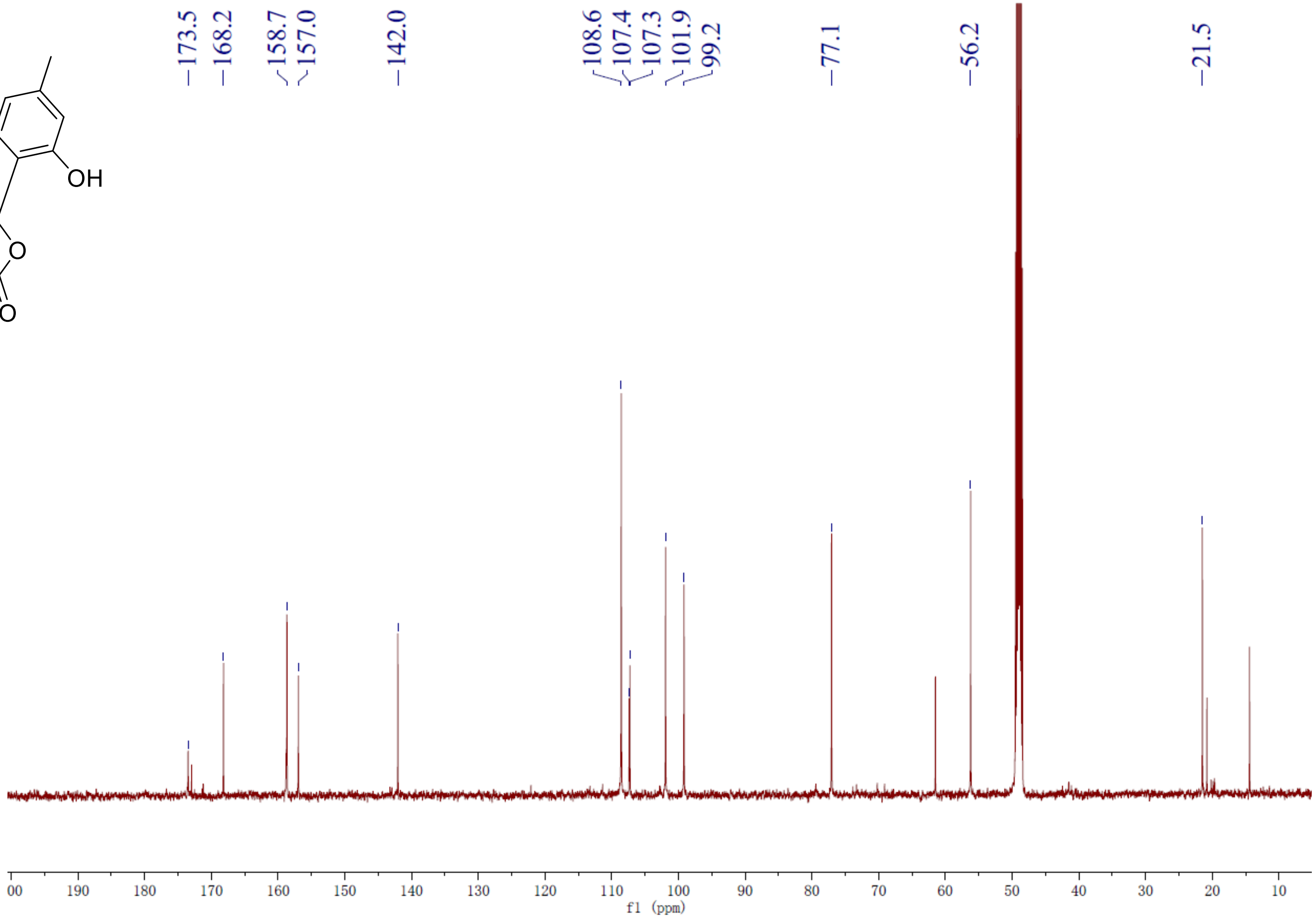

Figure S31. DEPT-90 °spectrum of compound **5** in mathanol-d_4 (125 MHz).

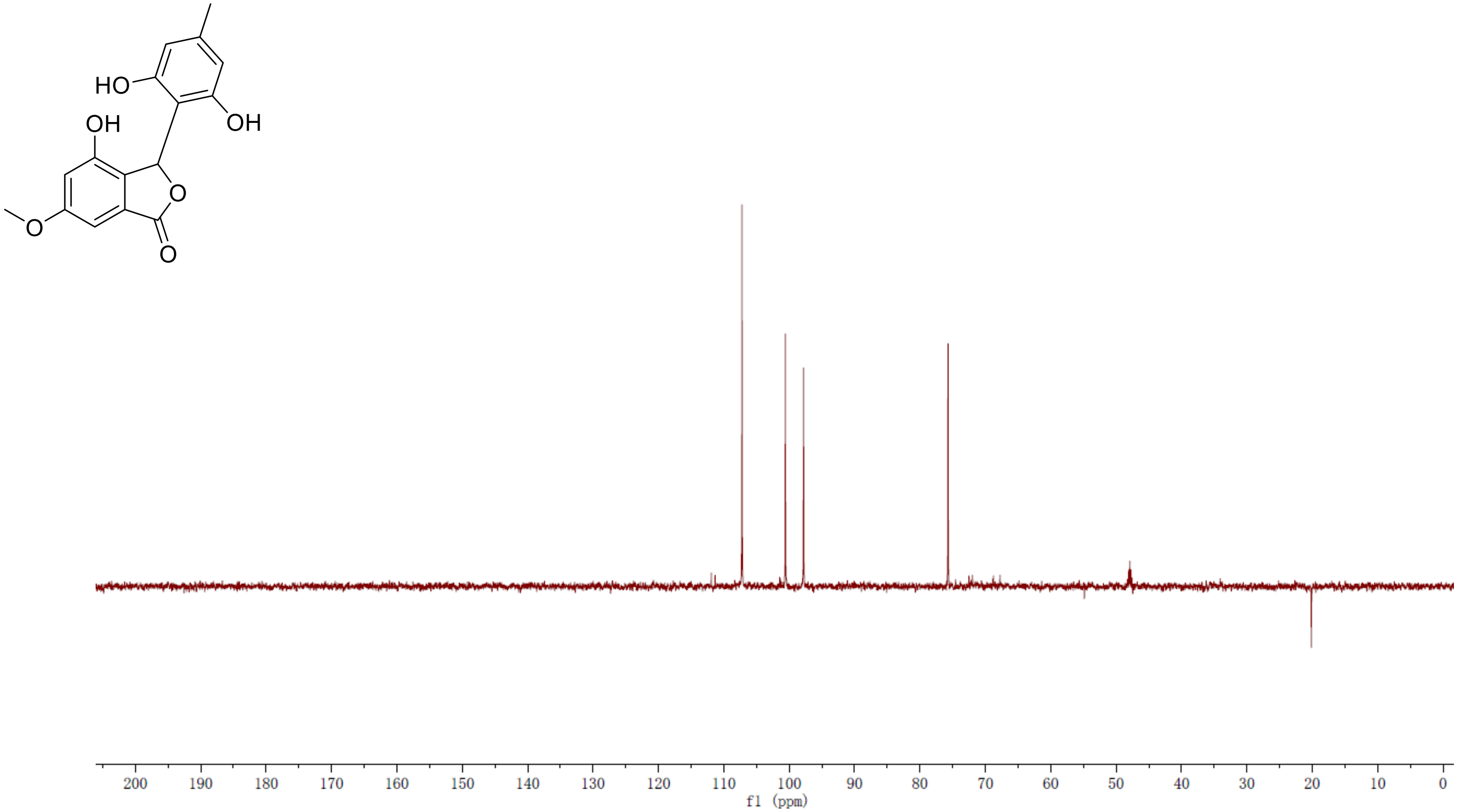

Figure S32. DEPT-135 °spectrum of compound **5** in mathanol-d_4 (125 MHz).

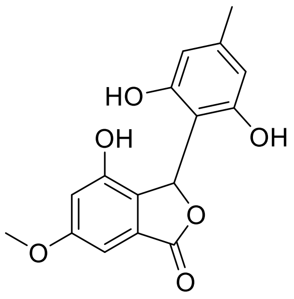

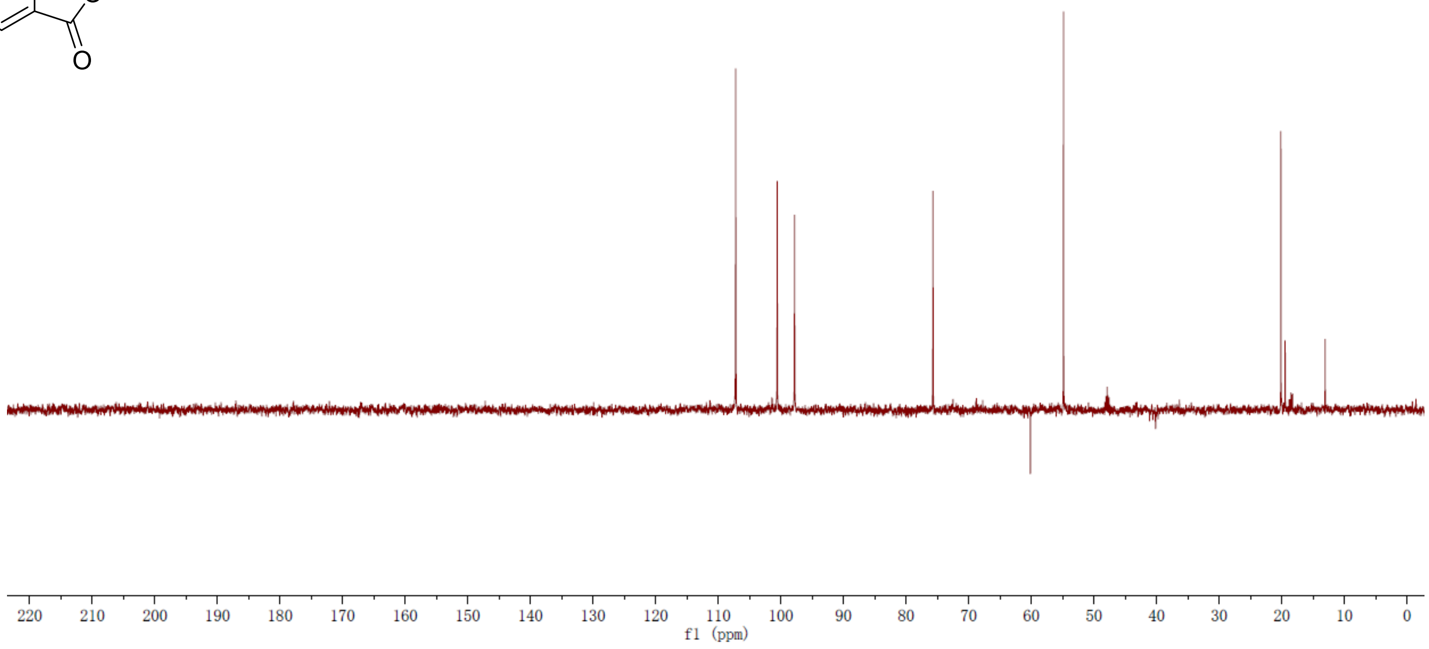

Figure S33. ^1H-^1H COSY spectrum of compound **5** in methanol-d_4 (600 MHz).

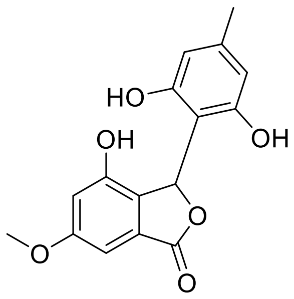

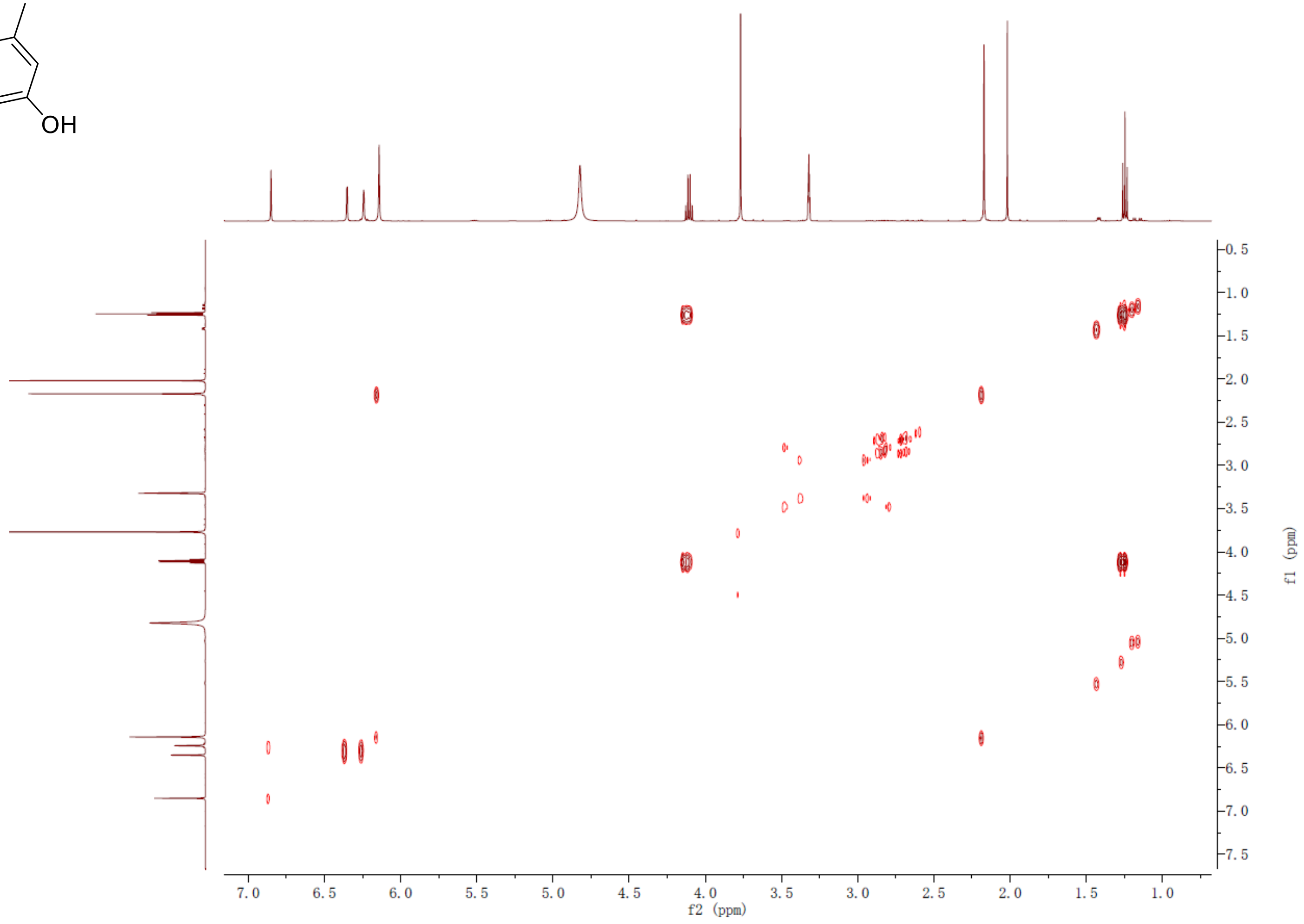

Figure S34. HSQC spectrum of compound **5** in methanol-d_4 (600 MHz).

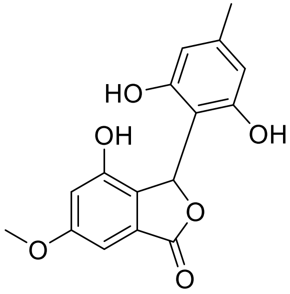

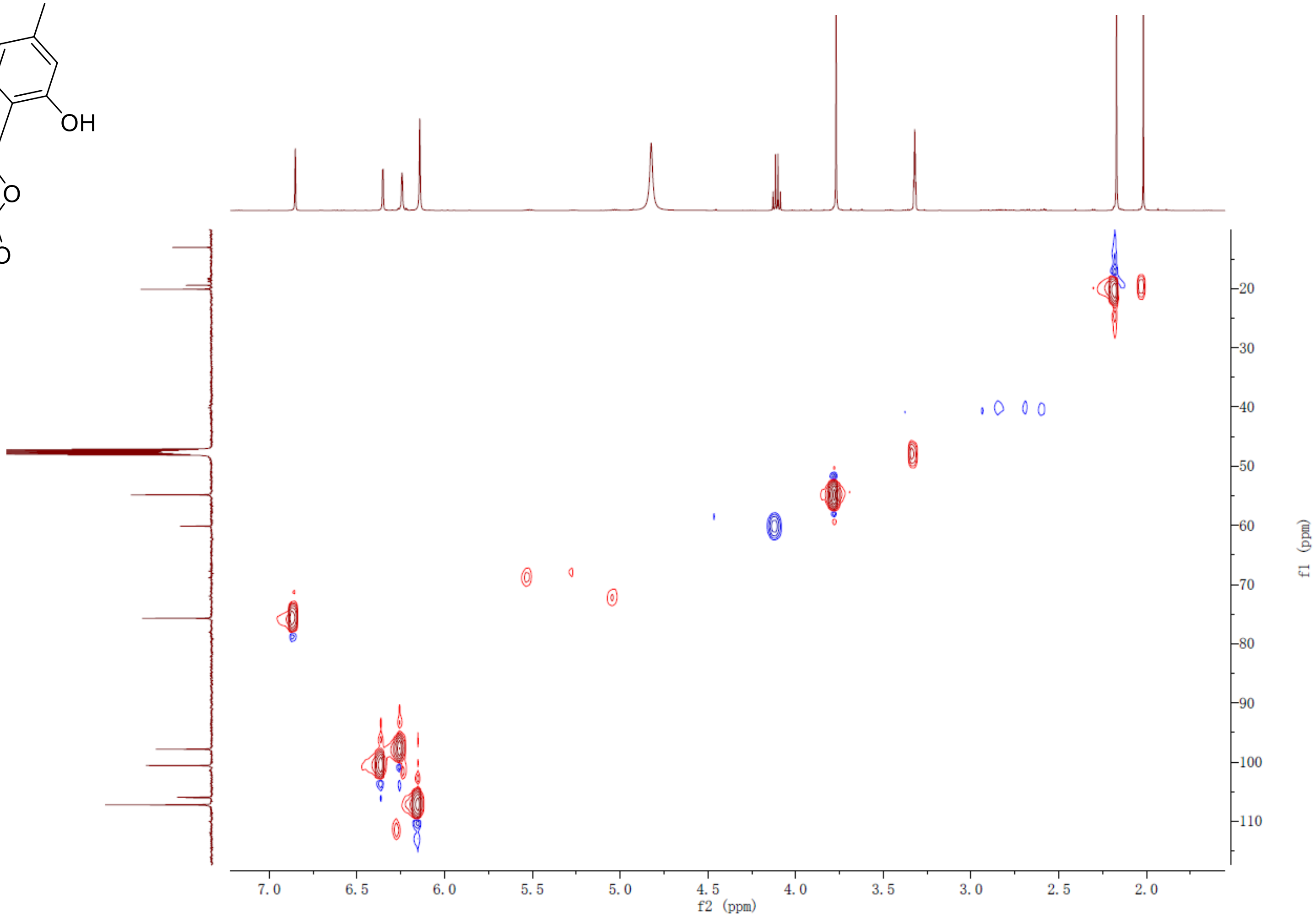

Figure S35. HMBC spectrum of compound **5** in methanol-d_4 (600 MHz).

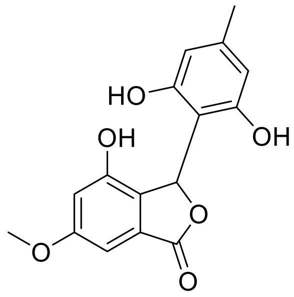

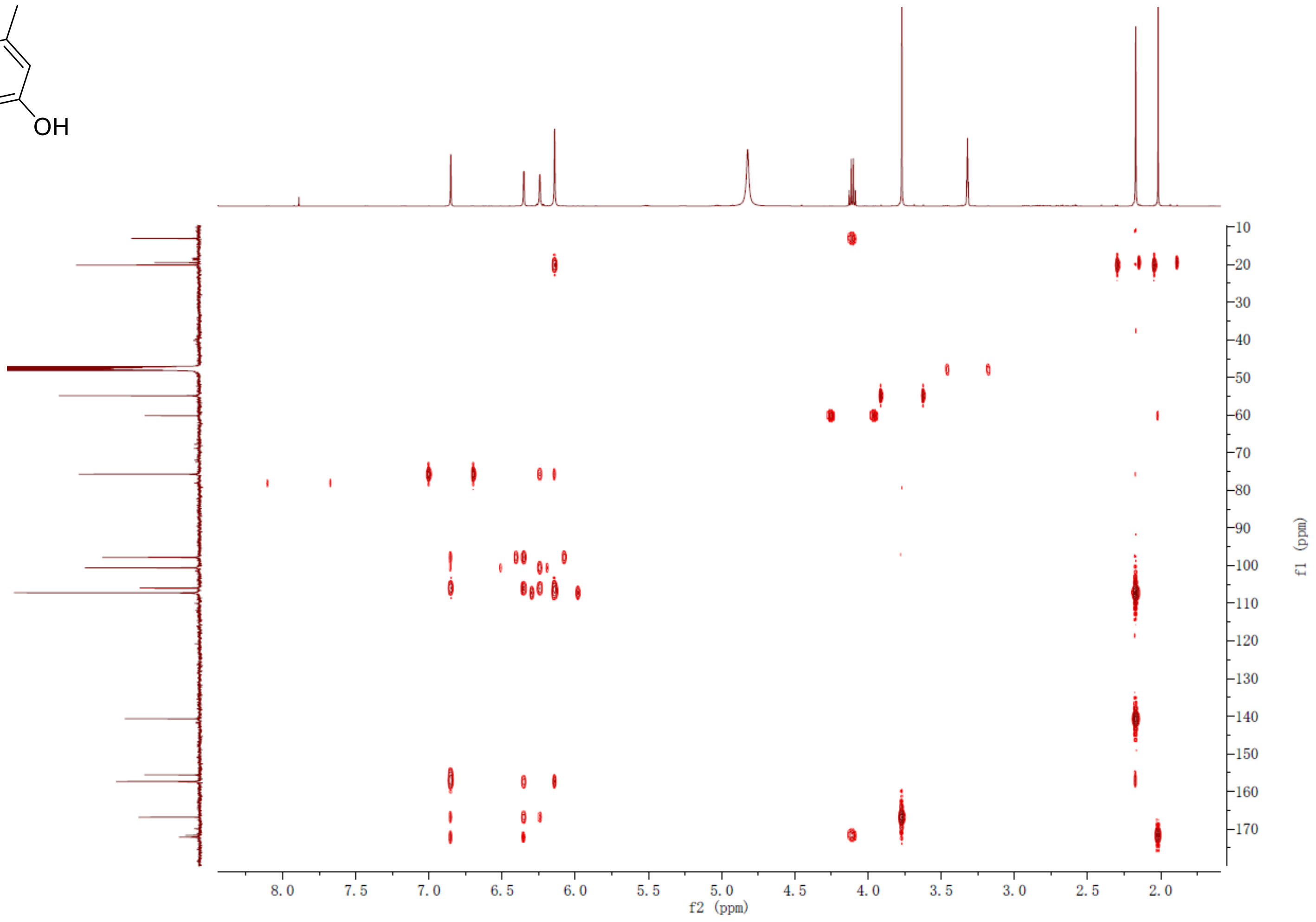

Figure S36. HRESIMS spectrum of compound **1**.

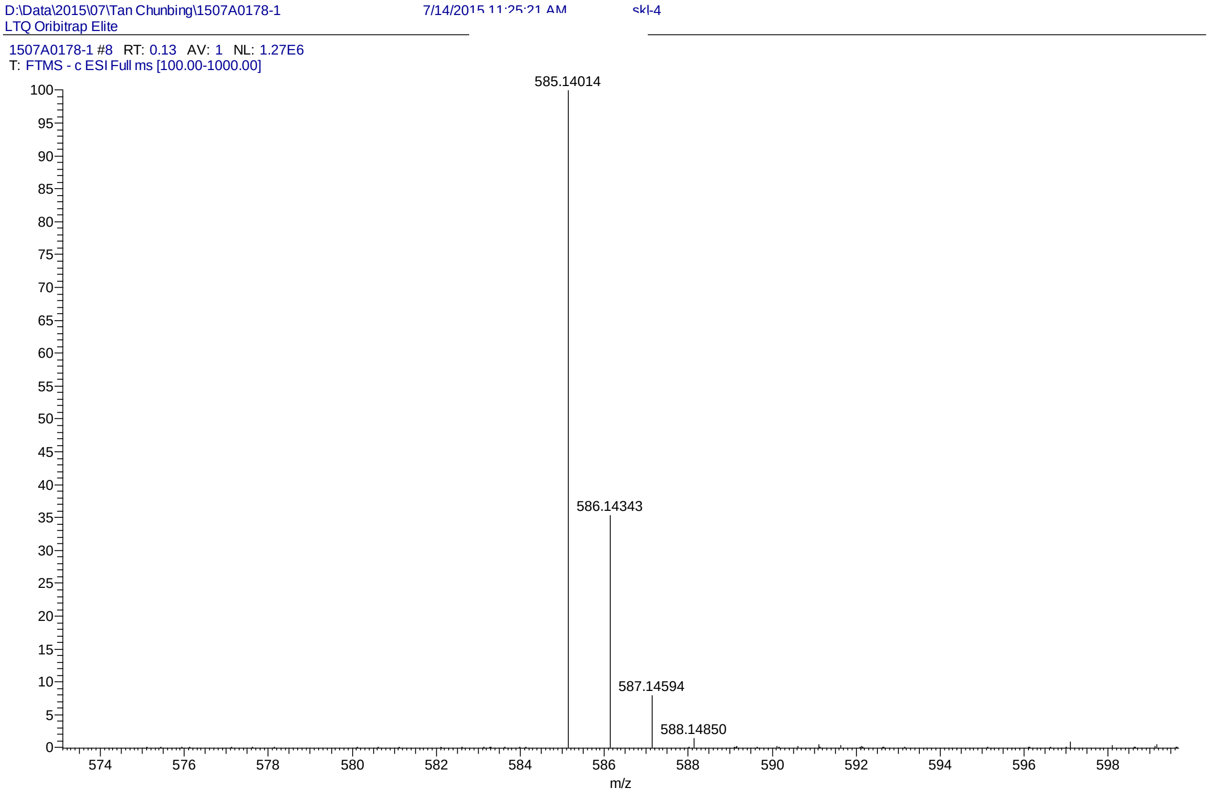

SPECTRUM - simulation :				
m/z	Theo. Mass	Delta (ppm)	RDB equiv.	Composition
585.14014	585.14023	-0.16	20.5	$C_{32}H_{25}O_{11}$

Limits:

(1) Charge: -1

(2) Nitrogen-Rule: Do not use

(3) Mass tolerance: 10.00 ppm

(4) Elements in use: ^{12}C (25~35), ^{1}H (0~60), ^{16}O (0~15)

Figure S37. HRESIMS spectrum of compound **2**.

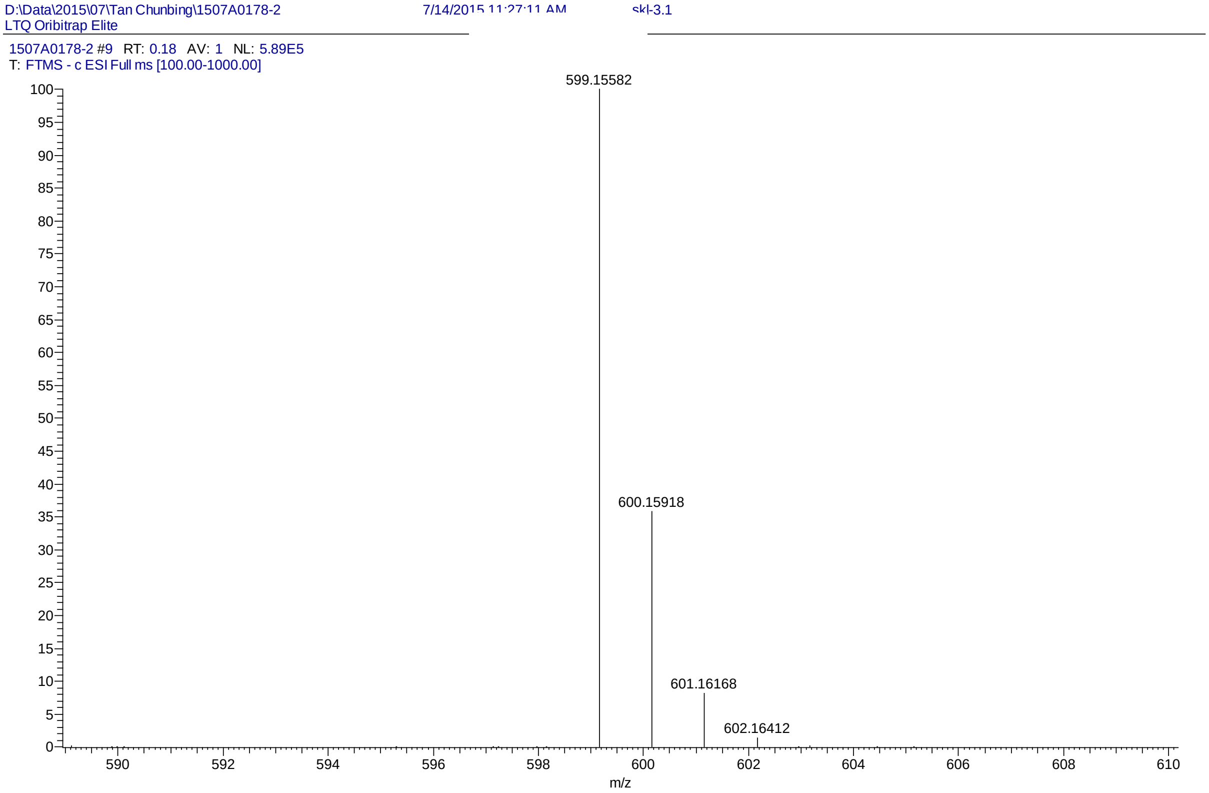

SPECTRUM - simulation :				
m/z	Theo. Mass	Delta (ppm)	RDB equiv.	Composition
599.15582	599.15588	-0.11	20.5	$C_{33} H_{27} O_{11}$

Limits:

(1) Charge: -1

(2) Nitrogen-Rule: Do not use

(3) Mass tolerance: 10.00 ppm

(4) Elements in use: ^{12}C (25~35), ^{1}H (0~60), ^{16}O (0~15)

Figure S38. HRESIMS spectrum of compound **3**.

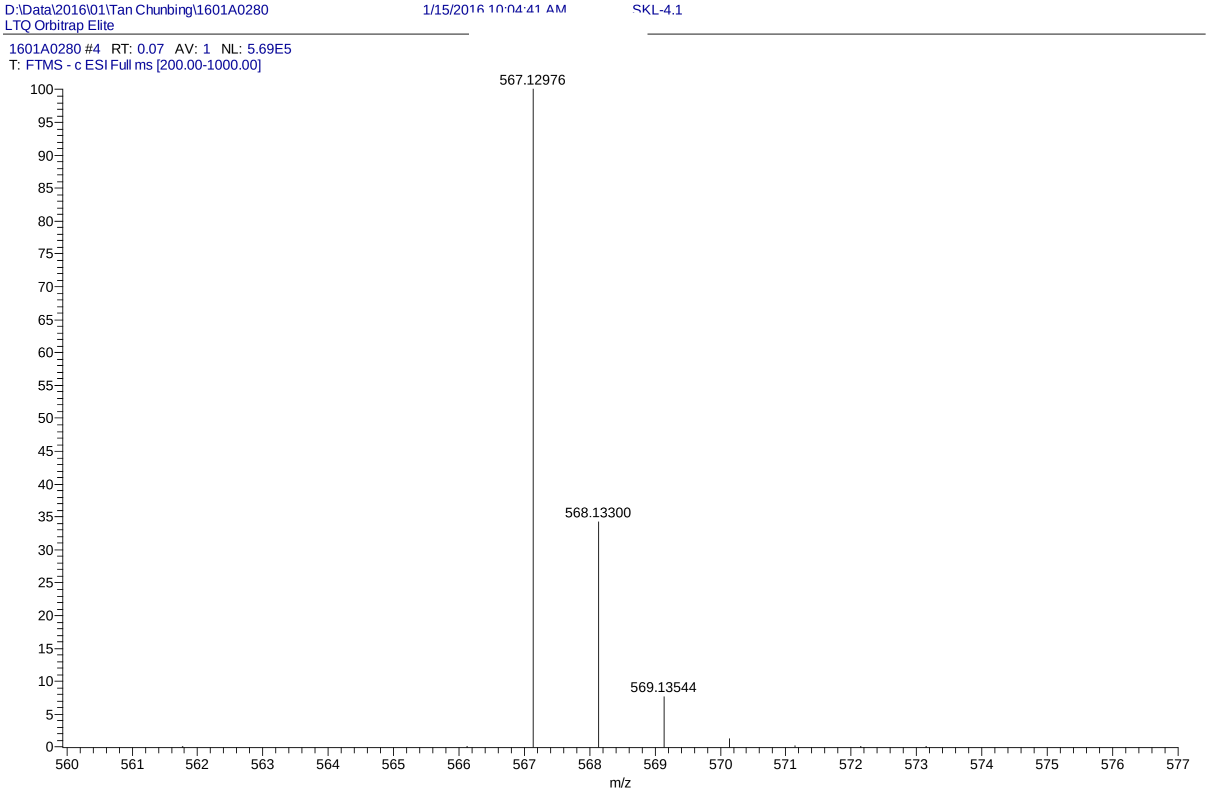

SPECTRUM - simulation :

m/z	Theo. Mass	Delta (ppm)	RDB equiv.	Composition
567.12976	567.12967	0.16	21.5	$C_{32}H_{23}O_{10}$

Limits:

(1) Charge: -1

(2) Nitrogen-Rule: Do not use

(3) Mass tolerance: 10.00 ppm

(4) Elements in use: ^{12}C (0~40), ^{1}H (0~60), ^{16}O (0~15)

Figure S39. HRESIMS spectrum of compound **4**.

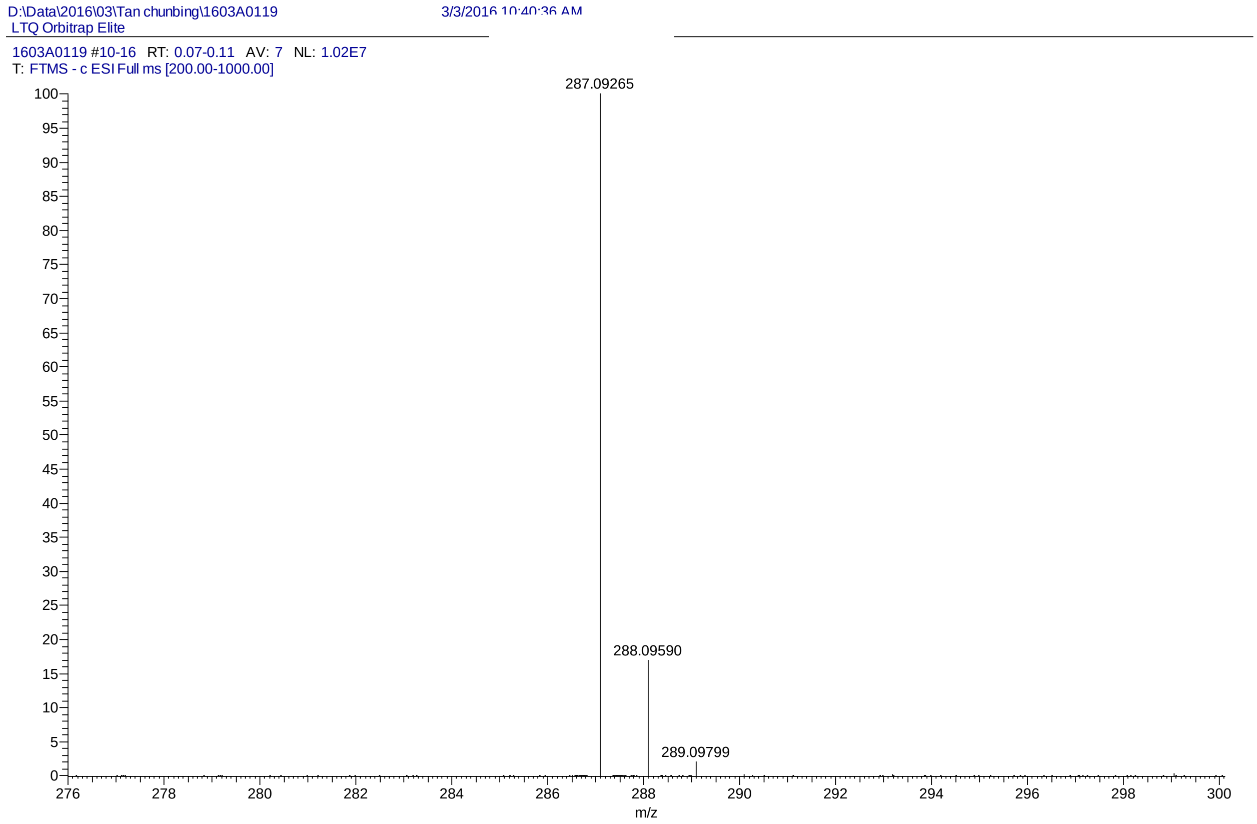

SPECTRUM - simulation :				
m/z	Theo. Mass	Delta (ppm)	RDB equiv.	Composition
287.09265	287.09250	0.53	9.5	C16 H15 O5

Limits:

(1) Charge: -1

(2) Nitrogen-Rule: Do not use

(3) Mass tolerance: 10.00 ppm

(4) Elements in use: ^{12}C (0~30), ^{1}H (0~60), ^{16}O (0~15);

Figure S40. HREI-MS spectrum of compound **5**.

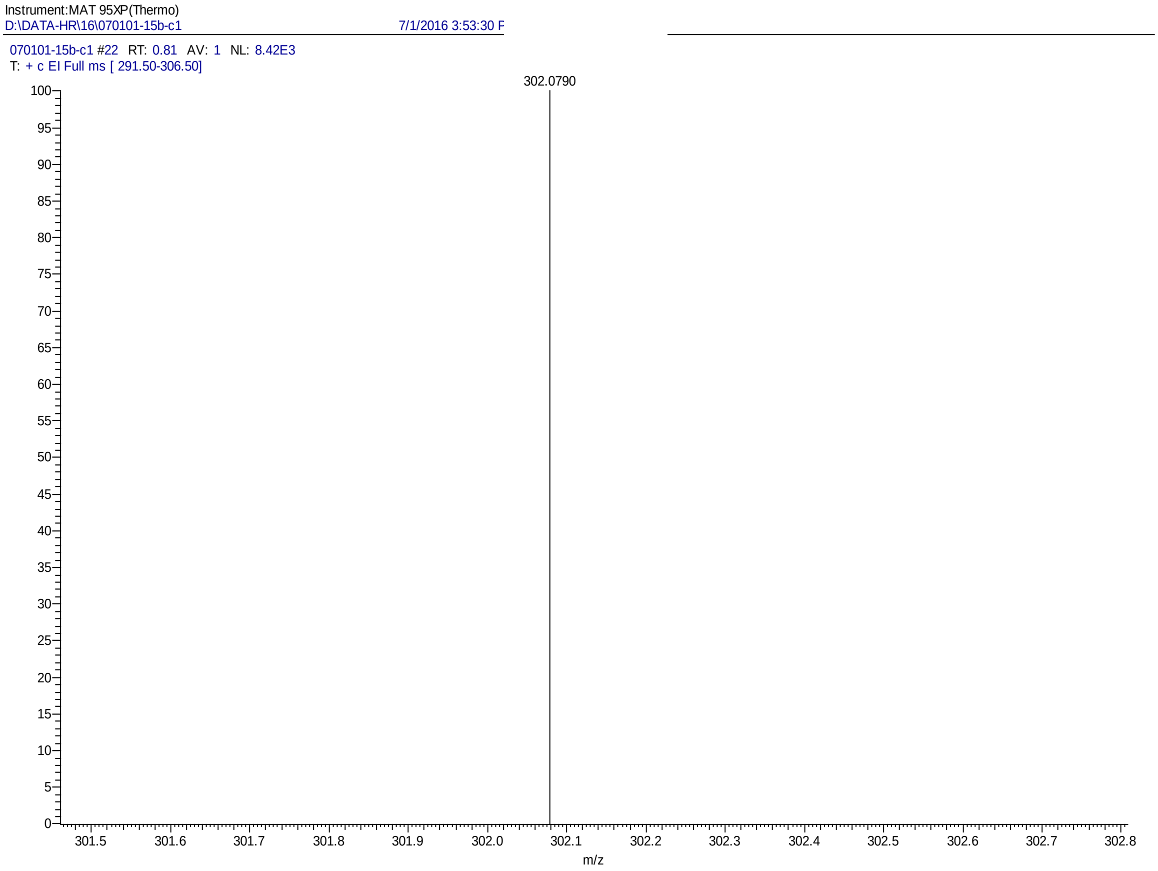

SPECTRUM - MS

File : D:\DATA-HR\16\070101-15b-c1.RAW

Full ms [291.500 - 306.500] - Range: 291.500 - 306.500

Scan No. 22 of 36

Scan #: 22

RT: 0.81

Data points: 1

m/z	Theo. Mass	Delta (ppm)	RDB equiv.	Composition
302.0790	302.0785	1.5	10	$C_{16}\,H_{14}\,O_6$
